# Refinement of Computational Access to Molecular Physicochemical Properties: From Ro5 to bRo5

**DOI:** 10.1021/acs.jmedchem.2c00774

**Published:** 2022-09-12

**Authors:** Matteo Rossi Sebastiano, Diego Garcia Jimenez, Maura Vallaro, Giulia Caron, Giuseppe Ermondi

**Affiliations:** † Molecular Biotechnology and Health Sciences Department, CASSMedChem, University of Torino, via Quarello 15, 10135Torino, Italy

## Abstract

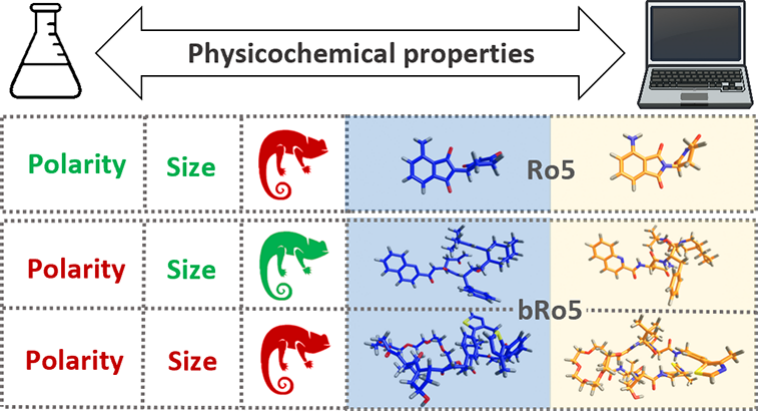

There is a need of computational tools to rank bRo5 drug candidates in the very early phases of drug discovery when chemical matter is unavailable. In this study, we selected three compounds: (a) a Ro5 drug (Pomalidomide), (b) a bRo5 orally available drug (Saquinavir), and (c) a polar PROTAC (CMP 98) to focus on computational access to physicochemical properties. To provide a benchmark, the three compounds were first experimentally characterized for their lipophilicity, polarity, IMHBs, and chameleonicity. To reproduce the experimental information content, we generated conformer ensembles with conformational sampling and molecular dynamics in both water and nonpolar solvents. Then we calculated Rgyr, 3D PSA, and IMHB number. An innovative pool of strategies for data analysis was then provided. Overall, we report a contribution to close the gap between experimental and computational methods for characterizing bRo5 physicochemical properties.

## Introduction

Classically defined drug-like molecules occupy the Rule-of-five (Ro5) chemical space defined by Lipinski^1^ and subsequently updated by other authors. Nevertheless, regardless of biologics and linear peptides, the number of orally bioavailable drugs and drug candidates with one or more Ro5 violations (bRo5) is constantly increasing,^2,3^ mainly including macrocycles and nonmacrocyclic compounds such as protein targeted degrading chimeras or PROTACs.^4^ These latter work by interacting with a protein of interest (POI) and a member of the E3 ubiquitin ligase complex: bringing the two in close proximity facilitates the ubiquitination of the POI, subsequently degraded by the proteasome machinery.^4^ PROTACs are extremely selective and potent, and their physicochemical profile is expected to identify a unique subset within the bRo5 space.^5^ Apart from one cyclic compound,^6^ all published PROTACs to date are linear, therefore no evident conformational restrictions are present.

bRo5 compounds are often affected by solubility/permeability and thus intestinal absorption and oral bioavailability issues.^7,8^ Therefore, to obtain new oral drugs in this space, molecular properties should be optimized in early drug discovery.^9,10^ Because property-based drug discovery can be applied at different development stages, both computed and experimental descriptors are required. Recently, we proposed a pool of experimental physicochemical descriptors suitable for quantifying lipophilicity and polarity for bRo5 molecules.^5,11^ Lipophilicity is efficiently determined using a series of chromatographic descriptors mimicking different environments. First, BRlogD relies on the measurement of the capacity factor at 60% acetonitrile using a XBridge Shield RP18 column.^12^ It was developed as a surrogate of the octanol/water partition coefficient (log *D* octanol/water).^12^ BRlogD works efficiently in the bRo5 chemical space, and it is crucial for the classification of PROTAC solubility.^7^ ElogD is another chromatographic log *D* octanol/water surrogate developed internally by Pfizer.^13^ Additionally, log *k*_W_^IAM^ implements an immobilized artificial membrane (IAM) column, which provides a lipophilicity index in an environment resembling the membrane phospholipids.^14^ Finally, log *k*′80 PLRP-S mimics the interior of the plasma membrane (nonpolar environment), being a surrogate of log *D* toluene/water.^15^ It is measured at 80% acetonitrile in a nonpolar polymeric chromatographic system named PLRP-S. Polarity can also be measured using chromatographic approaches. Δlog *k*_W_^IAM^, a descriptor obtained from the experimental log *k*_W_^IAM^ and BRlogD, was recently unveiled to model passive permeability of PROTACs.^16^ In addition, EPSA is another polarity descriptor based on a supercritical fluid chromatographic (SFC) system.^17^

Literature shows that bRo5 compounds may hide polar moieties in nonpolar environments and thus increase lipophilicity by lowering their polarity.^8^ This behavior, referred to as chameleonicity, is reputed to allow some compounds to become membrane-permeable.^18,19^ A chameleon is a molecule able to adapt to the environment by conformational adjustments. From the present definition, we assume that different conformer populations are adopted in water (expected to be more “open”), and nonpolar media (expected to be more “closed”). We also expect that the two conformer populations can interconvert. Chameleonicity is therefore a molecular property that deserves being quantified in bRo5 drug discovery programs. A possible experimental tracker of chameleonicity consists in using nuclear magnetic resonance (NMR) to verify that a given molecule exhibits conformers with different molecular properties in polar and nonpolar media.^20^ Unfortunately, few examples have been reported, probably because of the high level of expertise required to apply this methodology.^21,22^ ChamelogD, alias the difference between two chromatographic log *D* values obtained under different experimental conditions (BRlogD and ElogD), has been proposed by some of us as a simpler tool.^11^ Another interesting proposal suggests monitoring experimental chameleonicity through the analysis of the capacity factor in the previously described PLRP-S system.^16^ However, all of the methods are still poorly explored. Furthermore, we know that the formation of solvent-specific intramolecular hydrogen bonds (dynamic IMHBs)^23^ and other intramolecular interactions may drive chameleonic behavior. Experimental information about the propensity of compounds to form intramolecular hydrogen bonds (IMHBs) can be obtained from the combination of lipophilicity (e.g., the difference between log *P* measured in octanol and toluene, Δlog *P*_oct-tol_(24,27)) and polarity (EPSA^17^) data. Δlog *P*_oct-tol_ is a powerful tool that uses two different environments: octanol, in which IMHBs are not favored, and toluene, which promotes folded conformations, with IMHB formation when possible. Consequently, a low Δlog *P*_oct-tol_ suggests a higher propensity to form IMHBs, whereas the reverse is true for high values. Notably, this experimental descriptor has been validated by NMR. EPSA relies on polarity to assess the propensity of compounds to form IMHBs. Because EPSA is based on a polar stationary phase, less retained compounds (lower EPSA value) are less polar and thus have a higher propensity to form IMHBs.^17,24^

Despite these progresses in the experimental characterization of bRo5 compounds physicochemical profile, computational efforts are also needed to rank candidates for their potential as drugs in the very early drug discovery when chemical matter is still missing. Computed lipophilicity and polarity are often used to computationally drive hit/lead optimization. However, common log *P* calculators that are limited to the octanol/water systems have been mostly trained on Ro5 compliant compounds, thus failing with large and flexible compounds.^25^ Also the topological polar surface area (TPSA) alone is not sufficient for driving property optimization in the bRo5 chemical space.^18^ These failures are due to evidence that both 2D calculated log *P*/*D* (any method) and TPSA cannot catch the dynamic capacity to mask polar groups in nonpolar media and expose them in water to interact with the receptor.

Notably, chameleonicity may be hijacked as a medicinal chemistry strategy to simultaneously optimize solubility and permeability.^2^ Therefore, we reasoned that computational tools recapitulating not only lipophilicity and polarity, but also chameleonic properties are needed to be computed to get efficient property-based drug design. However, because experimental quantification of chameleonicity is feasible, but the strategy is case-dependent, and there is not a full agreement between published methods (for instance, data provided by crystallography,^18^ NMR,^20^ and ChamelogD^11^ do not always agree each other), no computational routine analysis can be generalized yet inside the bRo5 space to compute chameleonicity. One approach to this aim, reported in some papers,^22,26^ consists in computing the conformational ensemble of the investigated molecules and then monitor polarity and size/shape variation of biorelevant conformers present in polar and nonpolar media. Although appealing, a significant degree of uncertainty exists about which method should be used to compute a conformational ensemble, how to identify the biorelevant conformers, which molecular properties should be calculated and how.

Overall, a universal computational strategy focused on molecular properties and tailored to bRo5 compounds has not been yet defined. Therefore, the main aim of the paper is to provide guidelines for setting up property-based drug design strategies for bRo5 compounds. In practice, this is a starting point (and not a general conclusion) reporting about some relevant milestones deserving disclosure regarding the prediction of relevant molecular properties in the bRo5 chemical space, including chameleonicity.

**Figure 1:**
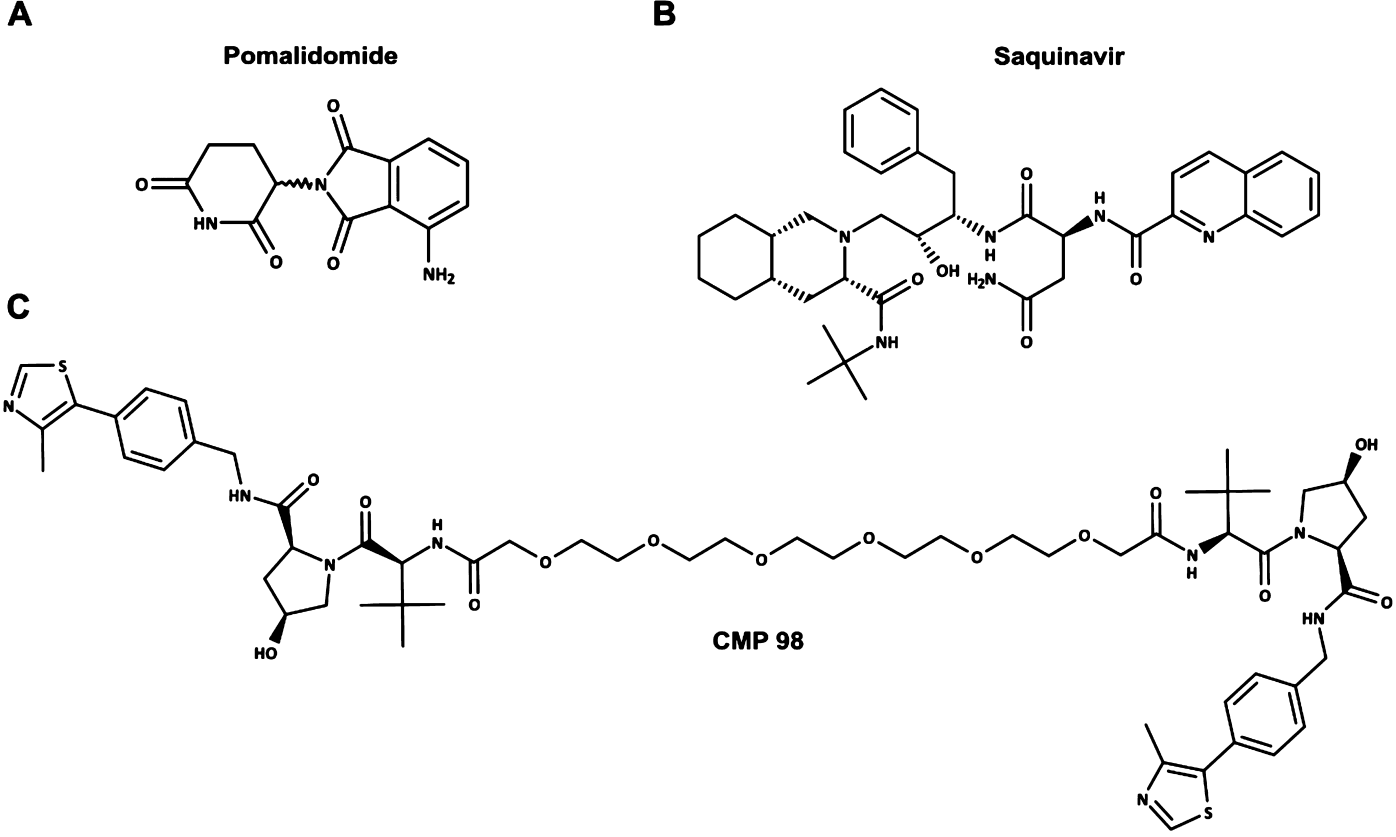
Structure of the three molecules selected: (A) Pomalidomide, (B) Saquinavir, and (C) CMP 98.

To reach the aim, we first selected three compounds representative of (a) Ro5 compliant space (Pomalidomide, Figure 1A), (b) bRo5 oral available drug (Saquinavir, Figure 1B), and (c) a polar, flexible, nonpermeable PROTAC (CMP 98, Figure 1C). The selection of the latter was made to represent a bRo5 compound included in the PROTACs chemical space^5^ that, unlike what the structure formula suggests, is not showing chameleonic behavior (see the Experimental Physicochemical Characterization section). To limit bias related to a different ionization profile, three compounds that can be considered neutral at physiological pH were chosen (Pomalidomide and CMP 98 are predominantly neutral in most pH range, and Saquinavir has a measured p*K*_a_ value of 7.1^18^ and thus it is mostly neutral at pH 7.4).

We are aware that an evaluation of computational methods would need a considerably larger data set, and an evaluation against an external set of molecules to verify the conclusions, but (as discussed above) the aim of the paper is to provide a starting point (missing in the literature) from which sound bRo5 property-based drug discovery design strategies can be set up.

In our opinion, predictions are reliable when validated with experimental data. Therefore, to setup a bRo5 drug design strategy based on computed molecular properties, in silico results should be validated with experimental lipophilicity, polarity, and chameleonicity data (see above). Therefore, the three compounds were submitted to an experimental analysis, providing the pool of physicochemical descriptors above explained. In particular, we measured lipophilicity in three different chromatographic systems (BRlogD, log *k*_w_^IAM^, and log *k*′80 PLRPS-S), we also determined two polarity indexes (EPSA and Δlog *k*_w_^IAM^) and the indicator of the capacity of the compounds to form IMHB, Δlog *P*_oct-tol_.^19,24,27^ Moreover, a qualitative index of chameleonicity (PLRP-S system) is also provided. As previously explained, most of these descriptors have been specifically designed for bRo5 drug discovery applications.^11^

Then, we moved to in silico strategies and for each molecule conformational ensembles in polar and nonpolar media were obtained with conformational sampling (CS) and molecular dynamics (MD) strategies. Next, ad hoc descriptors (3D PSA, Rgyr, IMHBs) were computed on each conformer after a revision of their significance and a careful selection of algorithms for their calculation. Given the different nature of CS and MD, we propose specific and different strategies to properly extract their information content. Infographic tools were extensively used to accomplish this task. The next step consisted in evaluating the role played by IMHBs in modulating polarity and Rgyr by monitoring representative conformers. Finally, we verified whether the in silico data could provide a similar information content of the experimental physicochemical data.

Overall, this study suggests how computational and experimental data obtained with ad hoc methods can be used to set up drug design strategies based on the properties of bRo5 compounds. The work also highlights the main problems that still exist in reaching definitive solutions. The use of only three compounds, far from leading to definitive solutions, is intended to lead to a broader discussion and a more detailed examination of the methodology.

## Results and Discussion

### Compounds Overview

This study focuses on Pomalidomide (Ro5), Saquinavir (bRo5), and CMP 98 (PROTAC). Pomalidomide (Figure 1A) is an immunomodulatory drug displaying antineoplastic activity. It acts by binding the E3 ubiquitin ligase complex component Cereblon, facilitating the degradation of the zinc finger transcription factor *Ikaros* and provoking downregulation of pro-inflammatory cytokines.^28^ Because of the selectivity for the degrading complex, Pomalidomide is one of the most used E3-binding building blocks in several PROTACs structures.^29^ Saquinavir (Figure 1B) is a protease inhibitor employed in the antiretroviral therapy and accepted as an oral drug. Saquinavir has been proved to be cell permeable, mainly by active transport and prone to P-gp mediated efflux.^30^ Independently from the contribution of the physicochemical profile to active transport mechanisms^8^ (beyond the scope of this work), we chose Saquinavir due to its chameleonic profile, contributing to a small but significant portion of passive permeation.^18^ CMP 98^31^ is a very flexible VHL-based homo-PROTAC, meaning it has the same E3 ubiquitin ligase complex binding moiety at both ends of the linker (Figure 1C) and mediates the self-degradation of the E3 complex.^31^ CMP 98 is not PAMPA permeable, but it is water-soluble (Supporting Information (SI), Table S1). Even if inactive and not displaying a favorable drug-like structure, the extremely high flexibility, the high number of IMHB acceptor, donor, and other polar groups (among which the long PEGylated linker) make CMP 98 a suitable model compound for challenging any hypothesis of chameleonic behavior in the bRo5 chemical space.

Seven common descriptors were recently revealed to be particularly useful in the early drug candidate characterization.^5^ They were computed for the three compounds (Table 1): nC (number of carbons) and MW (molecular weight) describe the molecular size, number of aromatic rings (NAR), the hydrophobic contribution related to the nonpolar region of the molecule, and TPSA, nHAcc, and nHDon (number of hydrogen bond acceptors and donors, respectively) provide a polarity estimate. Finally, PHI (Kier’s flexibility index) is included as a flexibility descriptor.

**Table 1:**
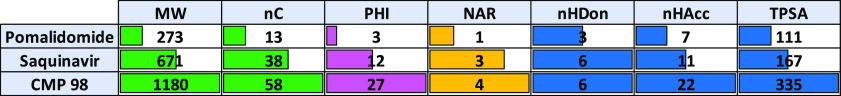
2D Molecular Descriptors of the Selected Molecules^a^

a Color codes account for: green (size), purple (flexibility), yellow (hydrophobicity), and blue (polarity).

Both MW and the nC atom show that CMP 98 is larger than Saquinavir, in turn larger than Pomalidomide, as evident from the structure already. Moreover, CMP 98 is significantly more flexible and has a higher nHAcc groups (and thus TPSA) than the two drugs. Notably nHDon count does not exceed 6.

Overall, these data show that both Saquinavir and CMP 98 are bRo5 compounds and, at least in principle, their flexibility could allow the formation of IMHBs and other intramolecular interactions, and the existence of stable conformers with reduced polarity and increased lipophilicity. Cyclosporin A is an example of bRo5 drug showing this behavior, having a TPSA value of 278 Å^2^.^23,32^

In spite of this analysis, in the next section, we will see that just Saquinavir is experimentally verified to be a chameleon.

### Experimental Physicochemical Characterization

Pomalidomide, Saquinavir, and CMP 98 were submitted to a set of experimental techniques to fully characterize their physicochemical profile. Notably, more than one descriptor is needed in the bRo5 chemical space.^11^

Lipophilicity was experimentally determined in three different chromatographic systems (Table 2): BRlogD^12^ refers to octanol/water (calculated log *P* values were reported in Table S2), log *k*′80 PLRP-S^15^ is a surrogate of log *P*_tol_ and log *k*_w_^IAM33^(33) refers to a chromatographic system in which phospholipids are immobilized on a silica-based support. Higher values of BRlogD correspond to more lipophilic compounds, as in the case of log *P*. The same trend is expected for log *k*_w_^IAM^, which is also often correlated with log *P* for neutral compounds. Finally, log *k*′80 PLPR-S is a chromatographic index in a reverse phase system, and thus the more lipophilic the compound the more retained by the stationary phase and the greater the *k*′ (and log *k*′) values. According to these remarks, experimental data support that Saquinavir is the most lipophilic compound in any investigated system.

**Table 2:**
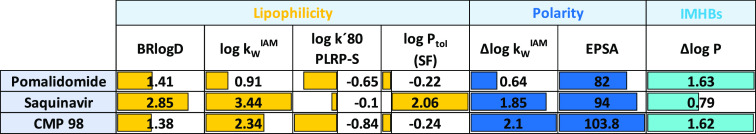
Experimental Descriptors of the Selected Molecules^a^

a Color codes account for yellow (lipophilicity), blue (polarity), and cyan (IMHB-formation capacity). SF: shake-flask method.

Polarity was quantified by two indexes (Table 2), EPSA,^17^ and Δlog *k*_W_^IAM^,^33,34^ the higher their values, the more polar the compound.^17,33,34^ Data reveal that CMP 98 is more polar than Saquinavir and far more polar than Pomalidomide.

Δlog *P*_oct-tol_(24) (the difference between log *P*_oct_ and log *P*_tol_) is an indicator of the capacity of a compound to expose HDon groups, and thus it quantifies the molecular capacity to form dynamic IMHBs. In practice, high value of Δlog *P*_oct-tol_ suggests the exposure of HDon groups and thus a limited capacity of the compound to form IMHB in nonpolar systems (Δlog *P*_oct-tol_ > 2.5 can be roughly assumed as an indicator of the absence of IMHBs). Our data support that Saquinavir is the compound with the highest capacity to form IMHB, its Δlog *P*_oct-tol_ being 0.79. Pomalidomide and CMP 98 show poorer propensity to mask HDon groups, although not negligible.

**Figure 2:**
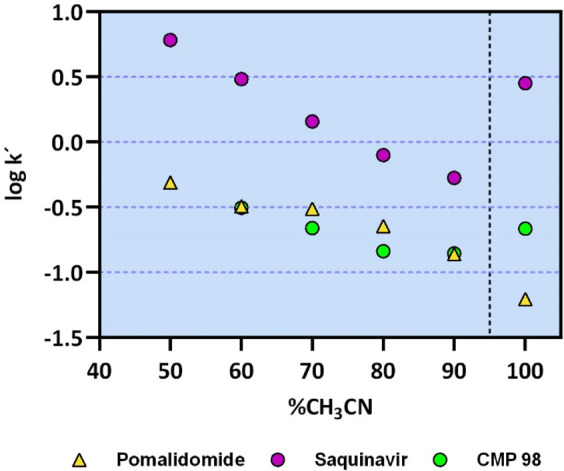
Pomalidomide (yellow), CMP 98 (green), and Saquinavir (violet) behavior in the PLRP-S system. The gray dashed line at 95% CH_3_CN highlights the slope change.

In a recent paper, we proposed to monitor the propensity of compounds to assume a chameleonic behavior through the analysis of the variation of the capacity factor in a nonpolar polymeric chromatographic system, named PLRP-S.^16^ Here we applied the same approach and determined the retention of the three compounds in the PLRP-S system with various mobile phases (from 50 to 100% ACN). According to the reverse-phase (RP) nature of the system, the retention time (and thus log *k*′ PLRP-S) of lipophilic molecules is expected to decrease when increasing the amount of acetonitrile in the mobile phase. Data are shown in Figure 2. The Pomalidomide behavior is about in line with expectations (yellow triangles). Notably, log *k*′ PLRP-S value at 100% cosolvent of Saquinavir (purple dots) is significantly higher than expected. This experimental finding can be explained by a propensity of Saquinavir to change its conformation in a nonpolar environment, masking its polarity supporting the chameleonic properties of this compound. For CMP 98 (green dots), some deviation from linearity is found, although significantly less pronounced than for Saquinavir.

Overall, the experimental data reveal that CMP 98 has lower lipophilicity and higher polarity than Saquinavir. CMP 98 also shows a low propensity to form IMHBs and a modest likelihood to behave like a chameleon, and ultimately a low ability to mask its polarity in response to environmental changes. These data are expected to be responsible for its low permeability (SI, Table S1). In the next sections, we explore whether similar information can be obtained with a pure computational analysis starting from the compounds’ chemical structure.

### Computational Strategies to Monitor Molecular Properties

As suggested by some of us,^26^ focusing on an ensemble of conformers in two environments (polar and nonpolar) and on physicochemical properties, represents a valuable approach to capture molecular behavior in the bRo5 space.

#### Conformers Generation

Among the plethora of available tools to generate conformers population, we selected three different methods. The first one is the conformational sampling (CS) built-in tool of the commercial suite Schrödinger/Macromodel (www.schrodinger.com). It is a hybrid method selecting between torsional and low vibrational modes^35^ based on the force field OPLS_2005.^36^ We chose water and chloroform as polar and nonpolar environments using an implicit treatment. We chose this approach because it represents a gold standard for medicinal chemists.^35^

We then performed a short (10 ns) molecular dynamics (MD) simulation because recent studies suggest that MD could be suitable for bRo5 molecules.^37^ The last approach consists in a steered molecular dynamics (SMD) protocol. SMD is an MD simulation, where an additional directional velocity term is applied to a subset of atoms.^38^ We speculated that if this is applied to the solute molecule, this will cross the periodic cell boundaries several times, simulating a continuous movement across the solvent molecules and putatively exploring a wider portion of the conformational space. We named this method SMD tunneling. For both MD and SMD tunneling, the CHARMM36m^39^ force field was employed, and the two explicit solvent systems were water and toluene. The choice of toluene was made because this solvent is experimentally used (e.g., for Δlog *P*_oct-tol_ determination).^24^ More details are reported in the Materials and Methods section.

It should be highlighted that CS and MD strategies provide independent information, and their comparison can be only made in terms of the final information content. In practice, we cannot compare conformations provided by CS and MD/SMD tunneling because the methods differ in at least three main aspects: (a) the applied force field (OPLS_2005 vs CHARMM36m, b) the solvent treatment (implicit vs explicit), and c) minimization or not of the conformers (just CS conformers are minimized). However, we can safely compare data provided by the two MD protocols.

#### Conformers Characterization by Molecular Properties: Descriptors Selection and Calculation Issues

In the previous section, we report lipophilicity and polarity experimental data for the three investigated compounds. Our aim is to find computational descriptors providing similar information.

In principle, we need to compute 3D lipophilicity in different environments. The most common 3D index of lipophilicity (named log *P*(MLP)^40^) is obtained from the molecular lipophilicity potential (MLP), which is calculated by projecting the Broto–Moreau lipophilicity atomic constants on the molecular surface. Although very appealing, this method shows some issues related to fragment parametrization mainly related to ionizable groups. Moreover, it is limited to the octanol/water system and its application is just suggested in some specific situations, but it cannot be accepted as a general tool. Overall, we do not dispose of efficacious in silico tools to compute 3D lipophilicity in different environments, and thus we decided to focus on polarity, size/shape, and IMHB descriptors to monitor the physicochemical profile of the three selected compounds. Their full analysis is also expected to capture chameleonic properties.

A molecular polarity descriptor can be efficiently computed; however, we need to establish a default methodology to be applied in any bRo5 study. As discussed in a previous report by some of us,^41^ polarity is often quantified by the polar surface area (PSA). Although several methods have been suggested to calculate the 3D molecular polar surface area (3D PSA),^42^ they rarely agree with each other. The main sources of variability lay in the definition of polar atoms, atomic radii, and the type of surface (solvent-accessible or molecular). Choosing a consistent method for 3D PSA calculation is important in order to be able to compare it with the 2D topological PSA^43^ (TPSA), the standard in the Ro5 chemical space.^42^ TPSA is calculated as fragment contribution, and thus it can be taken as an index of the highest 3D PSA values within a conformational ensemble,^18^ but also in this case, several programs differently implement TPSA calculations. For these reasons, we aimed to search for comparable TPSA-3D PSA method pairs. According to SI, Table S10, we chose the values of TPSA calculated by the software AlvaDesc (www.alvascience.com/alvadesc/) and the 3D PSA calculated with Vega^44^ (http://www.vegazz.net/, probe radius 0 Å) for further comparison. It should be noticed that no solvent-accessible PSA definition was included because no corresponding TPSA method is available. Nevertheless, we cannot deny that solvent-accessible 3D PSA sometimes better explains behavioral changes of molecules.^18^

Rgyr is a 3D description of molecular size and shape widely used in the bRo5 chemical space. In detail, Rgyr is correlated with molecular volume and shape. Rgyr is easily calculated as the root-mean-square distance (RMSD) between the compound’s atoms and its center of mass. It has been suggested to be a better surrogate for molecular size than MW in passive permeability studies.^45^

The systematic prediction of IMHBs is far from being a trivial task because calculation parameters strongly impact the outcome. Different software implements different settings, and thus different results are provided. We decided to use settings included per default in Chimera: relaxation of 0.4 Å (bond distance) and 20° (angle between HDon and HAcc).^46^ The used algorithm considers length and angle pairs, optimized for each moiety.^46^ Notably, Chimera does not detect the IMHB in Pomalidomide (amino group and *sp*^2^ hybridized oxygen in the phthalimide ring, SI, Table S11 and Figure S1).

### Single Property Analysis

Five sets of polarity descriptors were generated (Table 3) for the three compounds in both environments. The first two refer to real conformers obtained from the different methods (CS, MD, and SMD tunneling): the conformer with the lowest 3D PSA (MinPSA) and the one with the highest 3D PSA (MaxPSA). The remaining two polarity descriptors were the average (AveragePSA) and median (MedianPSA) 3D PSA values of the whole ensemble and do not correspond to real conformers. Finally, the difference between MaxPSA in water and MinPSA in nonpolar solvent (ΔMax_p_ – Min_np_) was also calculated. Relative values are in SI, Table S12.

**Table 3:**
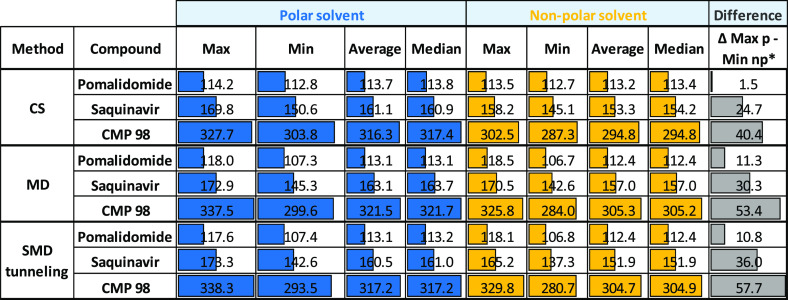
Statistical 3D PSA Values for the Generated Conformers with the Three Methods^a^

a *p, polar; np, nonpolar.

Table 3 supports that CMP 98 is always expected to be more polar than Saquinavir and Pomalidomide. ΔMax_p_ – Min_np_ suggests that CMP 98 varies its polarity the most when passing from a polar to nonpolar environment, especially in SMD tunneling ensembles. Our previous analysis on TPSA/3D PSA pairs shows that not all methods are directly comparable, thus setting sharp thresholds for cell permeability as done by Veber and co-workers (140 Å^2^)^47^ seems questionable. Indeed, Saquinavir and other bRo5 molecules are not systematically reaching this limit and still undergo passive permeability,^18^ showing that this threshold is not appropriate anyway. However, the lowest 3D PSA value of CMP 98 (281 Å^2^) remains sufficiently high to suggest that this represents a major limitation for passive permeability, in agreement with the experimental data.

**Figure 3:**
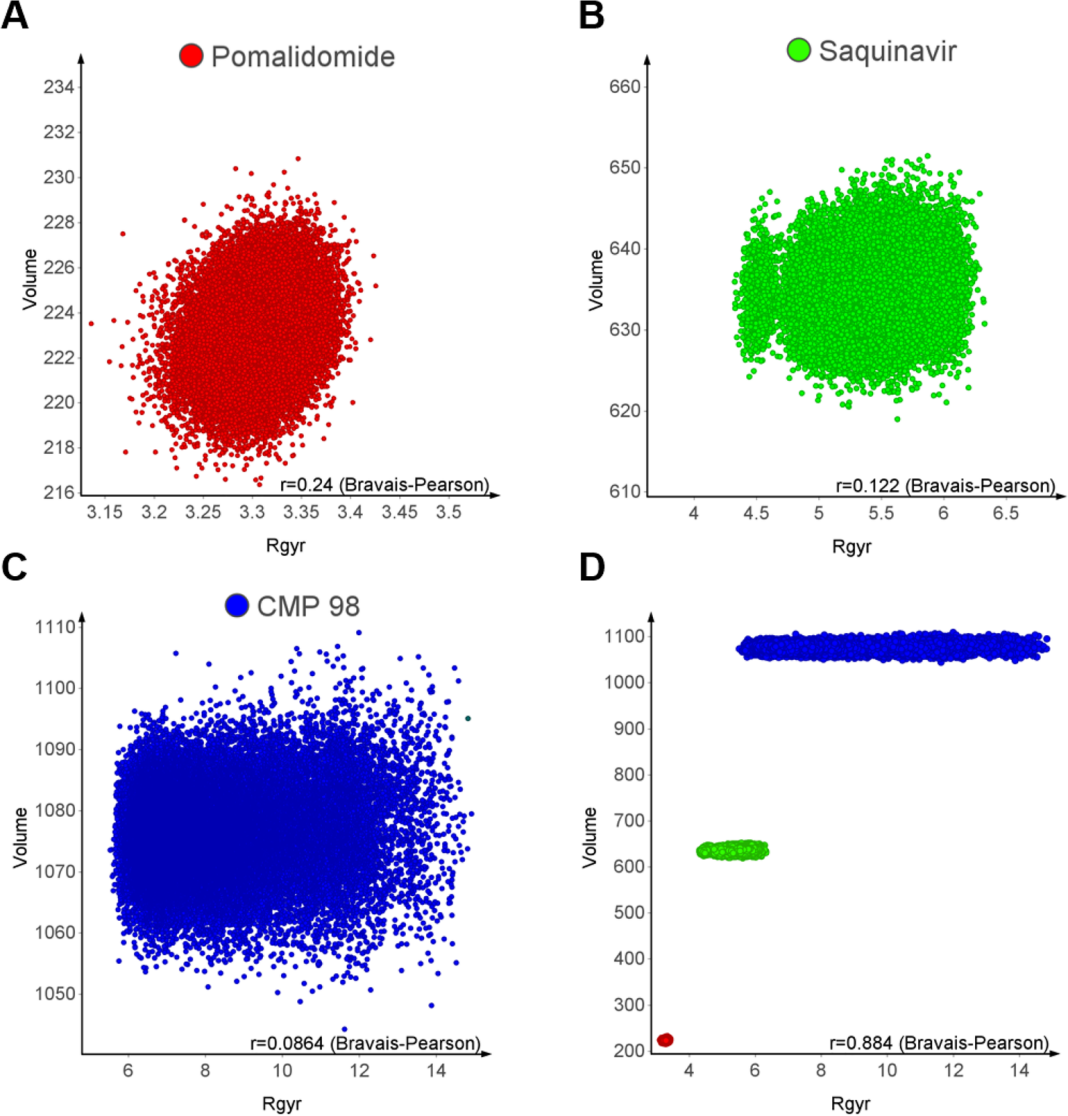
Correlation of molecular volume to Rgyr for (A) Pomalidomide, red, (B) Saquinavir, green, and (C) CMP 98, blue. (D) Conformational ensembles of the three molecules plotted together. Rgyr (Å) vs van der Waals volume (Å^3^). *R* is presented as Bravais–Pearson coefficient.

The second step of this analysis focuses on Rgyr. To clarify the radius of gyration information content, Rgyr was plotted against van der Waals volume (calculated with Vega, default conditions) for the three structures (Figure 3). As observed, there is a poor correlation between Rgyr and the molecular volume when the structures are individually considered, worsened with structure complexity. When the three structures are plotted together, Rgyr increases with increasing volume, but also the spread of Rgyr suggests that the two descriptors capture different properties. Therefore, even though Rgyr gives an idea of the size when different molecules are compared, in each compound’s conformational ensemblies, it should be rather considered as a descriptor of more or less elongated/spheric conformer shapes.

Table 4 corresponds to Table 3 when Rgyr replaces 3D PSA: the data suggest that (a) CMP98 has higher Rgyr than Pomalidomide and Saquinavir, according to the MW (Table 1) and (b) molecular shape varies considerably between the two environments (more elongated in water, more spheric in nonpolar media) for CMP98, at least when MD-SMD tunneling runs are investigated. Relative values are in SI, Table S13.

**Table 4:**
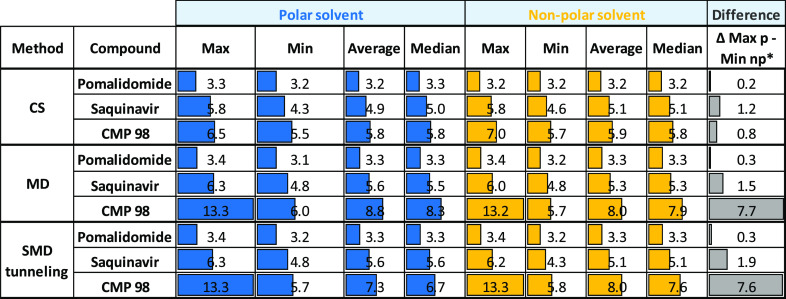
Statistical Rgyr Values for the Generated Conformers with the Three Methods^a^

a *p, polar; np, nonpolar.

**Figure 4:**
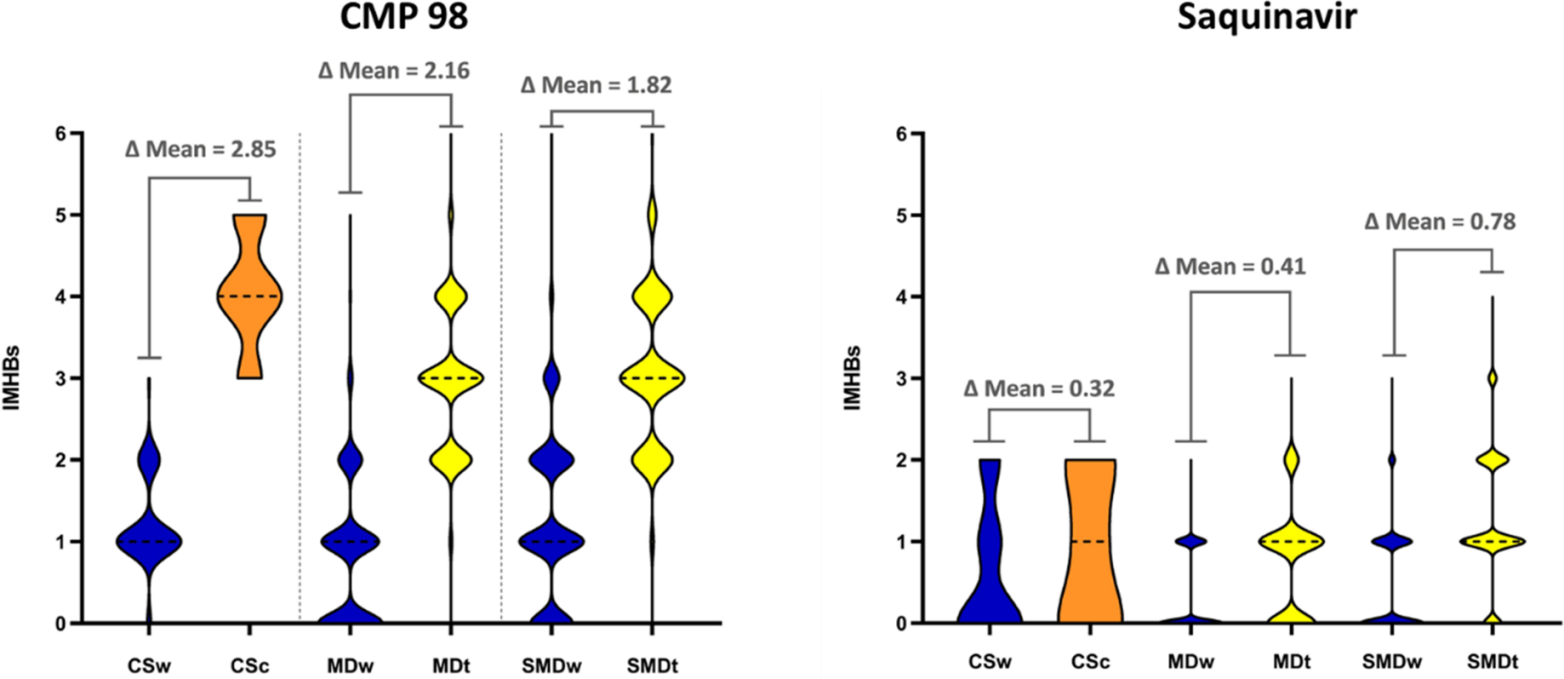
IMHB detection in CMP 98 and Saquinavir for conformational sampling in water/chloroform (CSw/CSc) molecular dynamics in water/toluene (MDw/MDt) and SMD tunneling in water/toluene (SMDw/SMDt). ΔMean is the difference of the average of IMHBs in nonpolar solvent and water. Median values are presented as black dashed lines.

Finally, we focused on counting IMHBs in the conformers from CS, MD, and SMD tunneling (Figure 4). As previously discussed, no IMHB was detected in Pomalidomide. CMP 98 displayed both the highest number and difference in IMHBs between the two environments (expressed as ΔMean IMHB in nonpolar/water (Figure 4)). Saquinavir, on the other hand, displayed fewer IMHBs than CMP 98 and lower ΔMean IMHB (nonpolar/water, Figure 4). Overall, such a monodimensional analysis of IMHBs consistently highlights a higher IMHBs formation capacity by CMP 98.

Overall, single property analysis suggests that CMP 98 in nonpolar media may at least in principle decrease its polarity and assume a more spheric shape. This is contradictory to the experimental data, thus we wondered if sole monodimensional analyses are adequate. The only point in agreement with the experiments remains the excessively high polarity of CMP 98, likely not enough to reach acceptable values for permeability. In the following sections, we performed more advanced combined property analyses to answer further questions.

### Bidimensional Property Analysis

In this section, we look at the jointed evolution of 3D PSA and Rgyr within the conformational landscape of our three model compounds. The use of these two descriptors is widely adopted in the literature, as discussed in the Introduction.

#### Conformational Sampling

The conformational behavior of Pomalidomide is not evidencing any significant property variation with the environment (SI, Figure S2), and its polarity can be well quantified with a 2D descriptor such as TPSA. Saquinavir and CMP98 show a different behavior than Pomalidomide because of their higher flexibility (Table 1).

**Figure 5:**
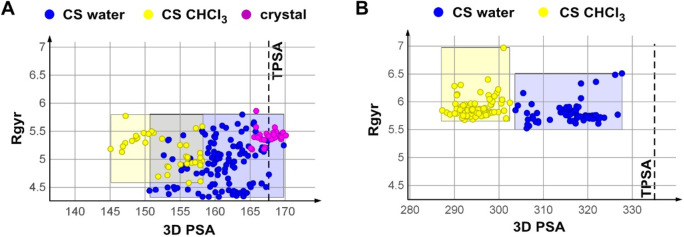
Conformational Sampling: 2D plot of 3D PSA vs Rgyr in water and chloroform: (A) Saquinavir and (B) CMP 98.

Rgyr is not able to distinguish conformers obtained in the two environments, although Figure 5A shows that the most spheric shapes are in water. A clear common trend instead locates chloroform conformers among the low 3D PSA-conformers. This result speaks in favor of solvent–dependent polarity separation, as previously suggested.^18^ Nevertheless, just Saquinavir water conformers reach the TPSA value (Figure 5A), suggesting that CMP 98 never exposes its full polarity (Figure 5B). Saquinavir, chloroform (yellow dots), and water (blue dots) conformers are partly superposed, as evidenced by the yellow and blue rectangles (Figure 5A). CMP 98 instead shows no superposition (Figure 5B), suggesting lack of stable conformers sharing common properties in both solvent systems. The presence of conformers with congruent properties in both solvents has been already suggested as a factor impacting on cell permeability of bRo5 drugs.^22^

Previous efforts to unravel dynamically solvent-exposed polarity was obtained by collecting X-ray resolved structures.^18^ At present, 0 for CMP 98 and 32 for Saquinavir are available: they fall within a high 3D PSA region superposed to TPSA-like water conformers (Figure 5A, purple dots). This tells us that Saquinavir crystallized from polar solutions (as in this case) is most likely to represent polar water conformers.

#### MD and SMD Tunneling

**Figure 6:**
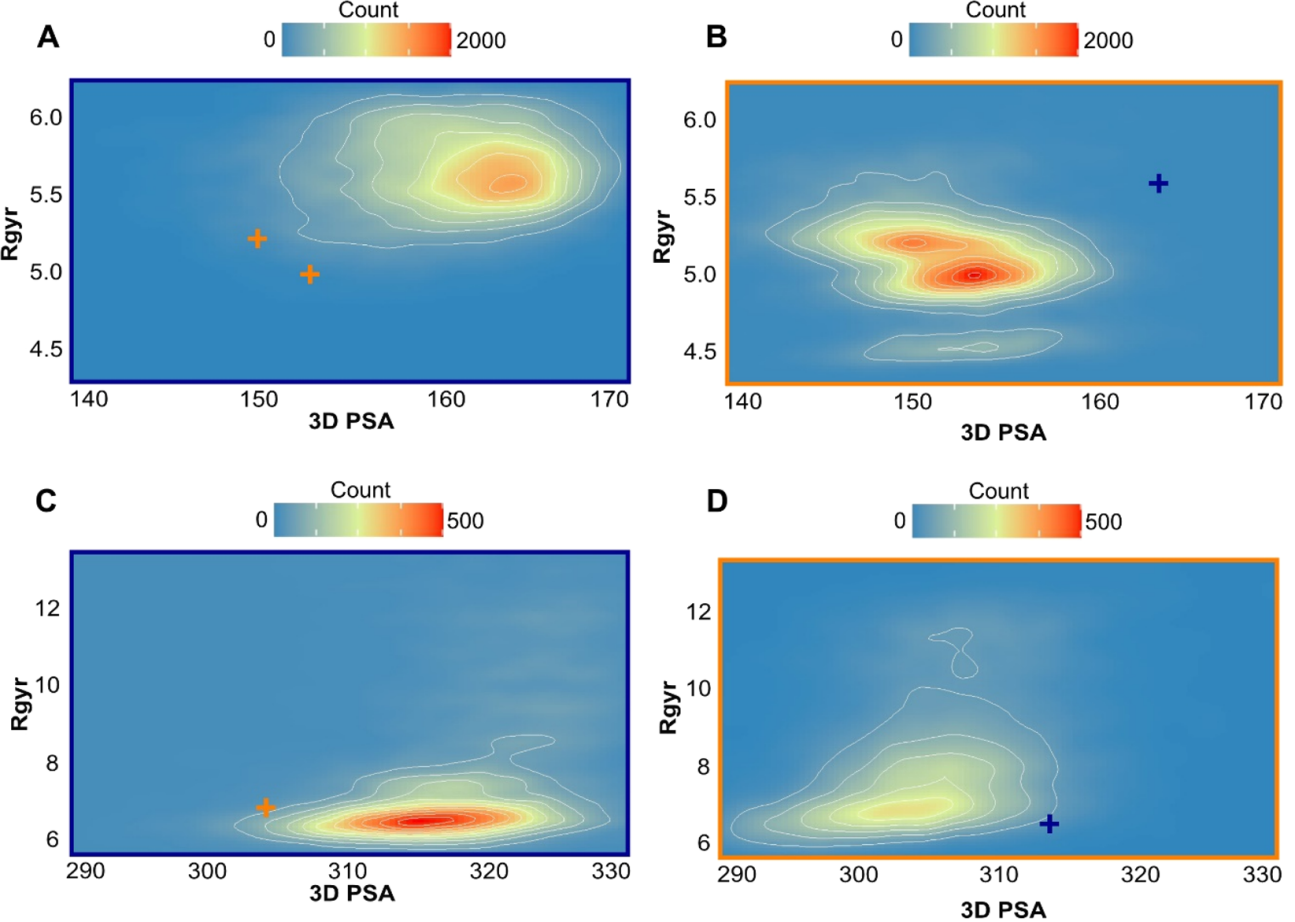
SMD Tunneling: density plots of Saquinavir in water (A) and toluene (B) and CMP 98 in water (C) and toluene (D) highlighting the dispersion patterns of the generated conformers in the 2D plot of 3D PSA vs Rgyr. The color scale is expressed as conformer frequency per tile. Blue perimeter stands for water, orange for toluene, and orange/blue crosses highlight the solvent-based shift of the inner cluster.

2D property density plots of MD and SMD tunneling simulations were built to monitor Rgyr and 3D PSA variation for conformers arising from molecular dynamics runs (SI, Figure S4 and Figure 6). Building and analysis of density plots is best suited to extract information from MD/SMD tunneling runs because the trajectories also include nonoptimized transient conformations.

The MD density plots of Saquinavir show high density clusters both in water (SI, Figure S4A) and toluene (SI, Figure S4B). CMP 98 instead shows no convergence to high density regions in water (SI, Figure S4C) and toluene (SI, Figure S4D), making any conclusion less definitive.

Property density analysis of SMD tunneling conformers reveal partially different patterns than those highlighted by MD, but also in this case a different behavior of the two compounds is highlighted. In particular, Saquinavir in water tends to occupy a slightly larger Rgyr region, (Figure 6A). In toluene, instead, two clusters (and not one, as in MD) are more populated than the outer regions (Figure 6B). CMP 98 water conformers are centered around a low Rgyr region, (Figure 6C) and the toluene ones result more dispersed, not individuating a high-density property region (Figure 6D, and SI, Figure S5B).

Overall, bidimensional analysis supports a different behavior between CMP 98 and Saquinavir independently of the tool used to generate conformers. We speculated that this could be related to a different propensity to form IMHBs. This will be described in the next section.

### Three-Dimensional Property Analysis

The next step of the data analysis consisted in moving to the simultaneous analysis of three molecular properties by integrating the investigation of IMHBs to the polarity and Rgyr monitoring.

#### Conformational Sampling

**Figure 7:**
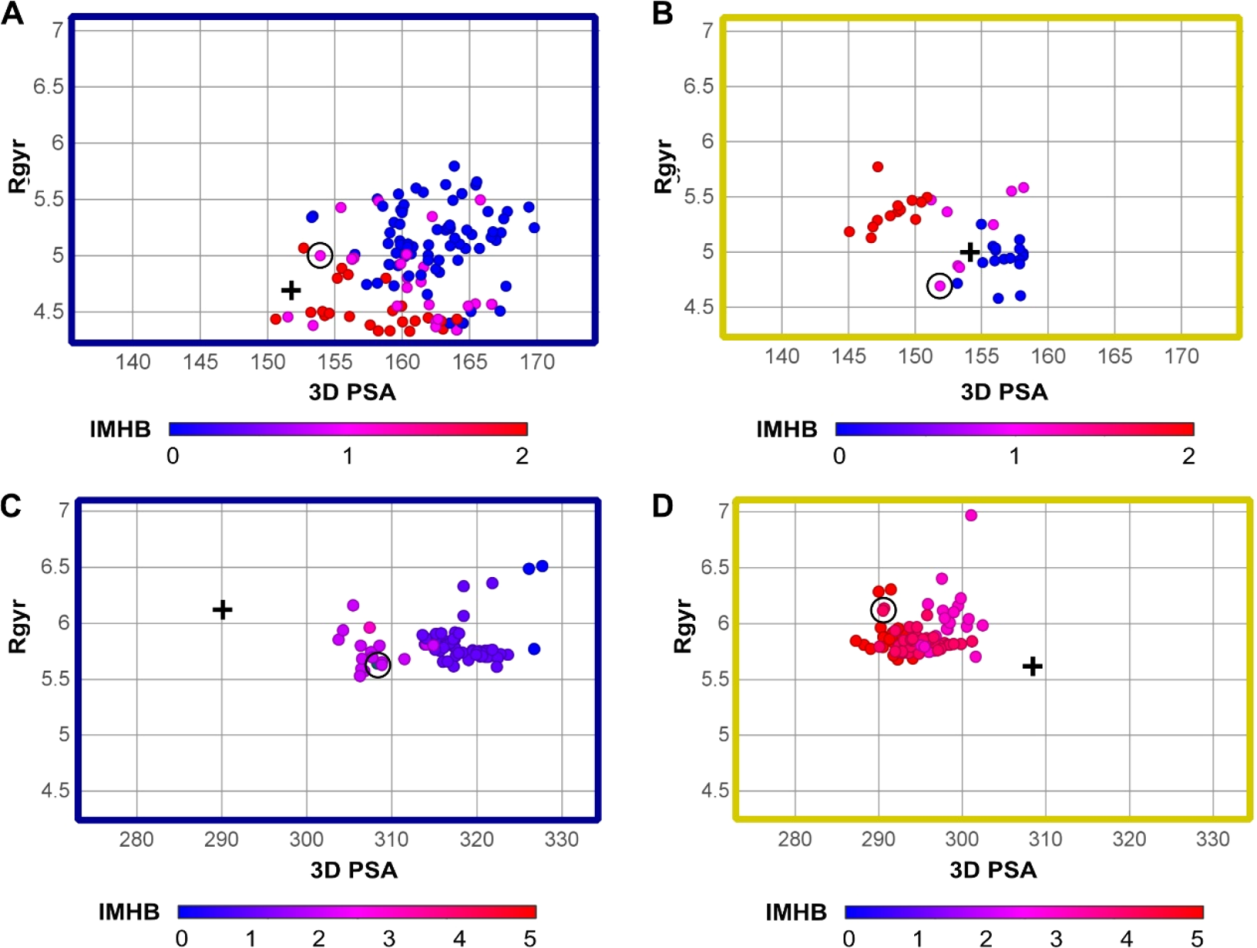
Conformational Sampling: distribution of IMHB regions based on size and polarity. (A,B) Saquinavir in water (A) and chloroform (B). (C,D) CMP 98 in water (C) and chloroform (D). Blue perimeter stands for water and yellow for chloroform. Black circles highlight the lowest energy conformer and black crosses the position of the correspondent low-energy conformer in the other solvent system.

In water, Saquinavir conformers with high number of IMHBs (2, red dots, Figure 7A) are not always characterized by low 3D PSA and low Rgyr, as expected. In chloroform, the separation by polarity is more striking, even though Rgyr shows the opposite trend (Figure 7B). Interestingly, the lowest energy conformers from both solvents, in principle the most abundant ones, show both 1 IMHB and similar 3D PSA values (Figure 7A,B, black circles/crosses).

CMP 98 instead reveals a different pattern: water conformers (Figure 7C) have mainly few IMHBs, whereas the opposite is true in chloroform (Figure 7D). Moreover, the polarity and IMHB separation of the two populations is supported by the lowest energy conformers (Figure 7C, D, black circles/crosses) displaying neither 3D PSA nor similar IMHBs count (respectively, 2 and 4 IMHBs in water and chloroform).

#### MD and SMD Tunneling

**Figure 8:**
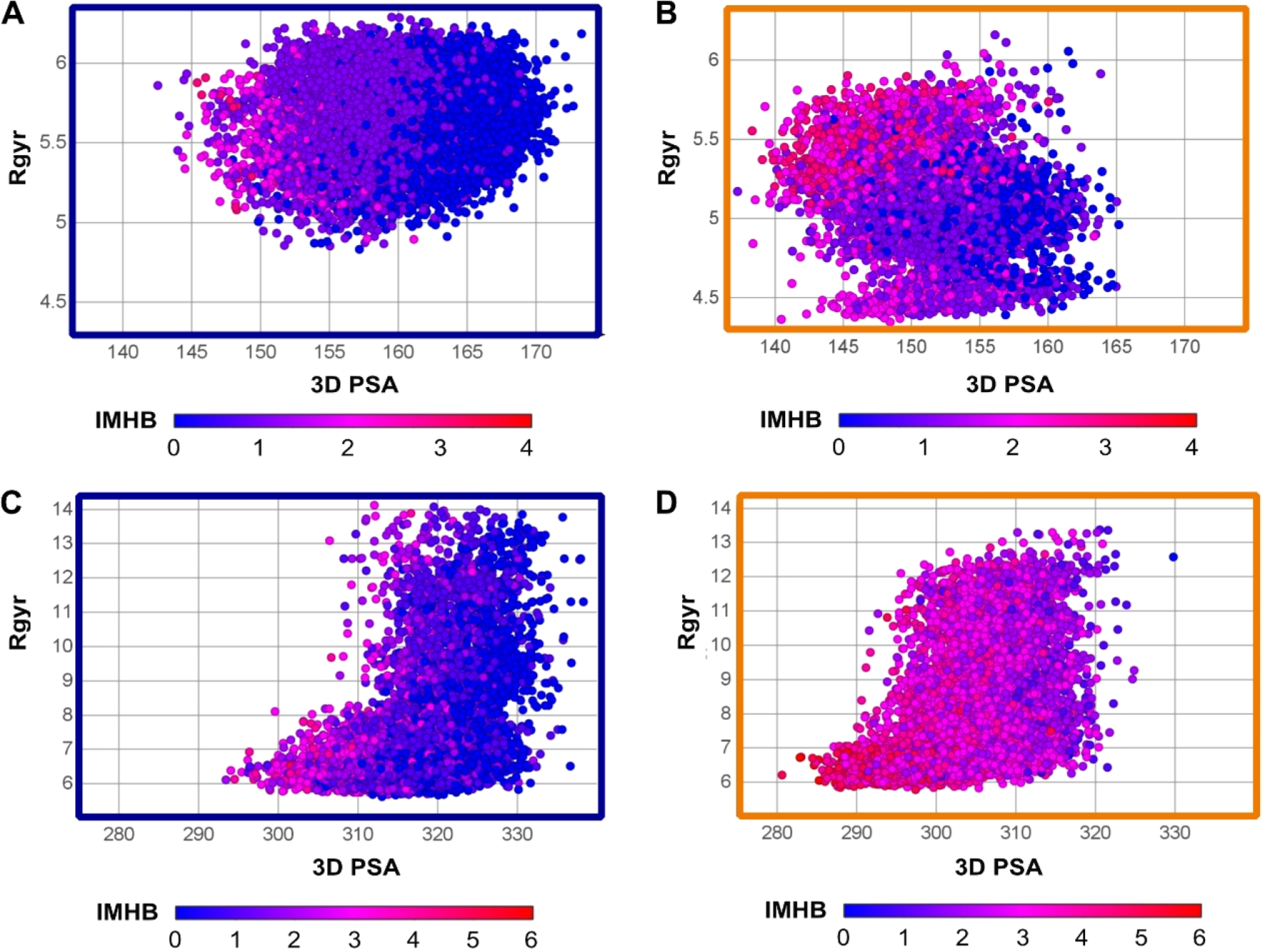
SMD Tunneling: distribution of IMHB regions based on size and polarity. (A, B) Saquinavir in water (A) and toluene (B). (C,D) CMP 98 in water (C) and toluene (D). Blue perimeter stands for water and orange for toluene.

The three-dimensional analysis of Saquinavir and CMP 98 conformers arising from MD and SMD tunneling in water and toluene depicts some differences (SI, Figure S6, and Figure 8) but highlights a common picture. In the case of SMD tunneling, both Saquinavir water (Figure 8A) and toluene conformers (Figure 8B) share a common subset of 1–2 IMHBs even though a higher proportion of conformers with more than 2 IMHBs is found in toluene (red dots). The conformers with more than 2 IMHBs often have lower 3D PSA than others. In turn, CMP 98 highlights most water conformers with 0 or 1 IMHBs (blue dots, Figure 8C) and toluene with 3 or more (pink/red dots, Figure 8D).

The conformers generated with MD (SI, Figure S6) have lower absolute number of IMHBs, but the overall pattern supports the SMD tunneling findings and thus reinforces the different role played by IMHBs in Saquinavir and CMP 98.

Overall, this analysis is in line with the bidimensional approach and supports the different behavior exhibited by Saquinavir and CMP 98.

### Selection and Investigation of Representative Conformers

**Figure 9:**
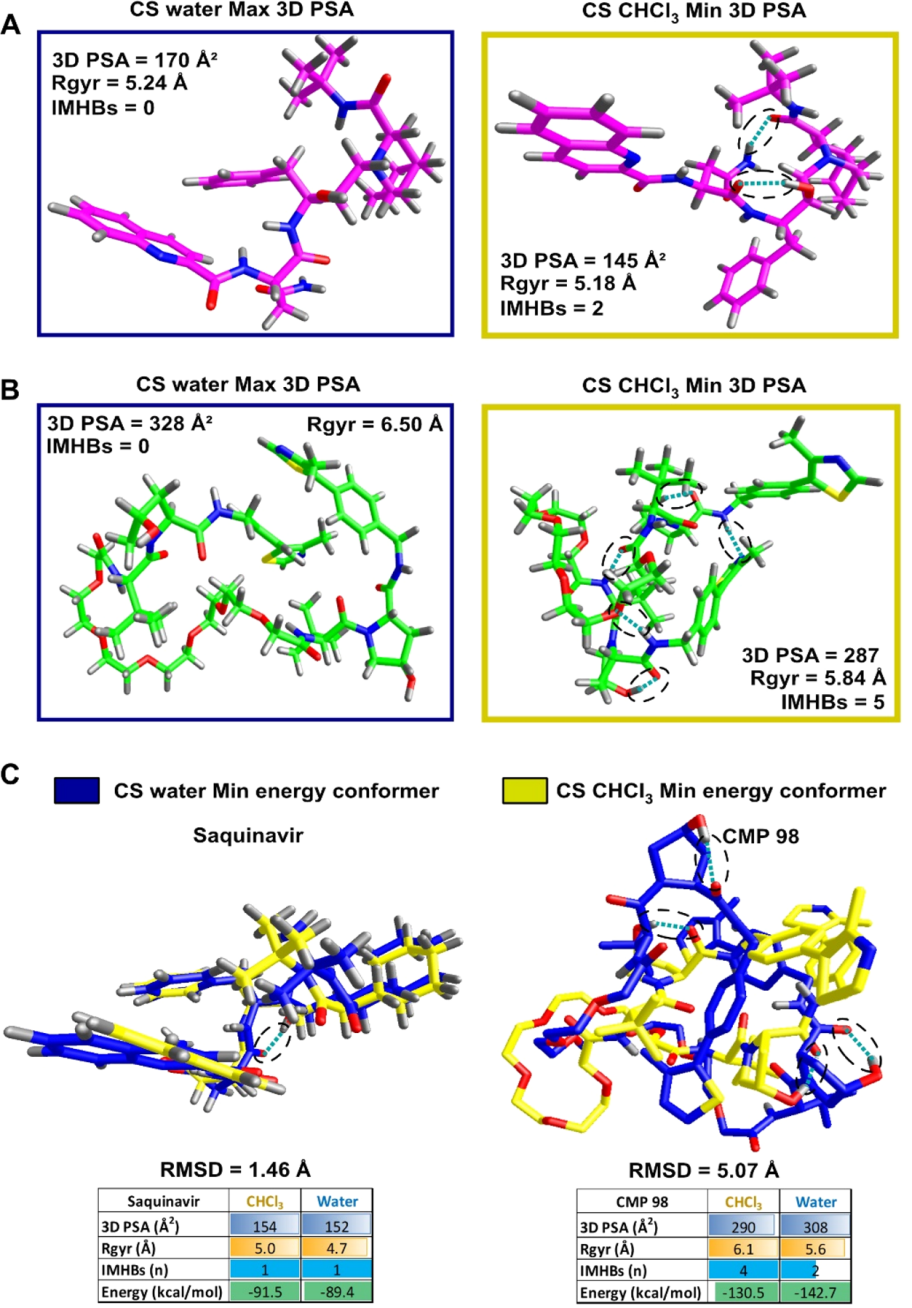
Conformational Sampling: conformers selection. IMHBs are depicted by dashed ovals. Blue perimeter stands for water and yellow for chloroform. (A) Saquinavir (purple) conformers corresponding to Min and Max 3D PSA in water and chloroform. (B) CMP 98 (green) conformers selected upon 3D PSA in water and chloroform. (C) Superposition (heavy atoms) of minimum energy conformers in chloroform (yellow) and water (blue) for Saquinavir (left) and CMP 98 (right).

Being conformers from CS determined upon a selection of energy minima, it becomes evident that extreme high–low polarity pairs could represent a first valid strategy to safely explore the borders of such a conformational ensemble. 3D PSA extremes (Min in chloroform, Max in water) reveals a smaller 3D PSA, Rgyr, and IMHB variation by Saquinavir (Figure 9A) than by CMP 98 (Figure 9B).

Next, we focused on energy-minimum conformers. Low-energy water and chloroform conformers share similar properties for Saquinavir but not for CMP 98 (Figure 9C). This is backed by structural features, where conformers superposition reveals coherence in Saquinavir (RMSD = 1.4 Å) but not for CMP 98 (RMSD = 5.07 Å). This result suggests that exclusively focusing on energy-minimum conformers is not enough informative.

As far as MD and SMD tunneling simulations are concerned, the closest conformers to the center of the density clusters are expected to be of relevance in this context. We are aware that density mapping could be backed by higher theory-founded methods,^37^ but we envisioned that the computational expense and high level of expertise required might prevent their routine applications in early drug discovery.

The MD conformers closest to the center of the density clusters depict a situation where Saquinavir preferentially forms 1 IMHB in toluene and none in water (SI, Figure S7A). CMP 98 forms 1 IMHB in water and 2 in toluene (SI, Figure S7B). Interestingly, the acceptor–donor pairs are neighbors (within 4 bonds), without either long-range or linker-involving IMHBs (SI, Figure S7B). Moreover, the IMHB missing in the water conformer (SI, Figure S7B, lighter dashed oval) shows analogue heavy-atom distance. In practice, those representative support that Saquinavir has an exclusively dynamic IMHB pattern, while CMP 98 not.

**Figure 10:**
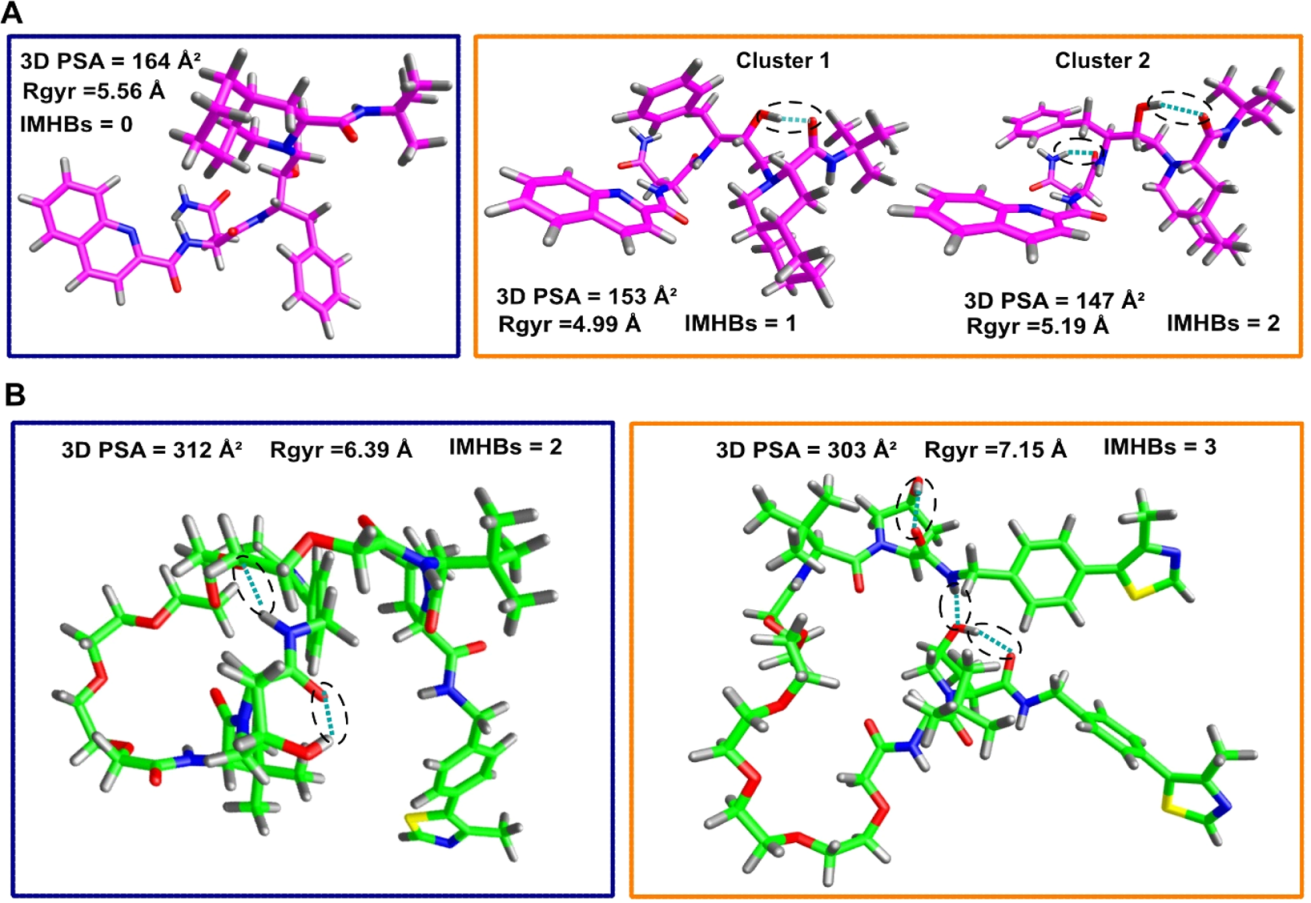
SMD Tunneling: conformer selection upon closeness to the center of the density plot (Figure 6). Blue perimeter stands for water and orange for toluene. IMHB are depicted by dashed ovals. (A) Saquinavir (purple). (B) CMP 98 (green).

Saquinavir conformers centered to the toluene SMD tunneling high density regions form either 1 or 2 IMHBs, while in water no IMHB is found (Figure 10A). Conversely, CMP 98 displays a more similar number of IMHB in water and toluene (3 and 2), with none involving the polar linker (Figure 10B). Interestingly, two IMHBs reveal their static nature, being conserved in both solvents and being formed between the same pairs in the γ position already found in the MD run (SI, Figure S7B).

Overall, this conformer analysis also shows a different behavior exhibited by Saquinavir and CMP 98 mainly related to the different nature (static vs dynamic) of their IMHBs.

### Computational and Experimental Data: Agreement and Missing Points

Experimental results showed that CMP 98 has lower lipophilicity, higher polarity than Saquinavir and Pomalidomide, poorer capacity of forming IMHBs, and behaves like a chameleon. Moreover, CMP 98 is soluble but not permeable. Thus, the experimental physicochemical profile as a whole suggests that Pomalidomide is a rather standard Ro5 drug, Saquinavir displays some features of molecular chameleons being the reason for its oral bioavailability and the reverse is true for CMP 98.

With regard to the computational approach, single analysis of each property (both 2D and 3D descriptors) highlights that the impact of flexibility is modest for Pomalidomide and that CMP 98 is the most polar of the three drugs. However, this analysis is not differentiating other aspects of CMP 98 and Saquinavir; at least in principle, they both seem to form more IMHBs in nonpolar environment and decrease their polarity, in disagreement with the experimental information.

More relevant information arises from the bidimensional analysis. To extract the full information content of the 3D PSA vs Rgyr plot based on CS conformers, we focus on three main aspects, potentially related to a chameleonic behavior: (a) if conformers obtained in different environments can be separated based on polarity/shape, (b) we look for superposition regions including congruent conformers in both environments, and (c) if conformers with the highest polarity have 3D PSA almost equal to TPSA. Results support that Saquinavir behaves differently from CMP 98 agreeing with a chameleonic profile. This is revealed by the evidence that Saquinavir shows less polar conformers in nonpolar media (with poor shape separation) with a certain degree of superposition between conformers obtained in water and chloroform. Moreover, there are Saquinavir conformers with a 3D PSA almost equal to TPSA. The density plots obtained from MD and SMD tunneling runs also suggest a certain degree of chameleonicity exhibited by Saquinavir because its conformers preferentially fall within defined property windows and this behavior goes along with the idea of a chameleonic molecule assuming a restricted number of closed conformers in nonpolar environments.

The addition of IMHB count as the third dimension shows that not all of them have the same effects on the molecule’s architecture. In fact, the formation of IMHBs in Saquinavir involves a polarity and shape variation, whereas in CMP 98, it does not. Explanatory evidence comes from the analysis of representative conformers where Saquinavir is more extended in water, not forming any IMHBs and converging toward conformers with lower 3D PSA and more IMHBs in a nonpolar environment (dynamic IMHBs). Moreover, even though not so clear, Rgyr seems to decrease when passing from polar to nonpolar media, suggesting an impact of IMHBs on molecular architecture. CMP 98 instead shows either nonconclusive results (MD) or an opposite pattern (SMD tunneling) with formation of some static IMHBs (= present in both environment), strongly reduced polarity variation, and the presence of more open conformers in the nonpolar environment.

Overall, different computational analyses show different degrees of adherence to the experimental evidence (Table 5), but the results as whole are in acceptable agreement.

**Table 5:**
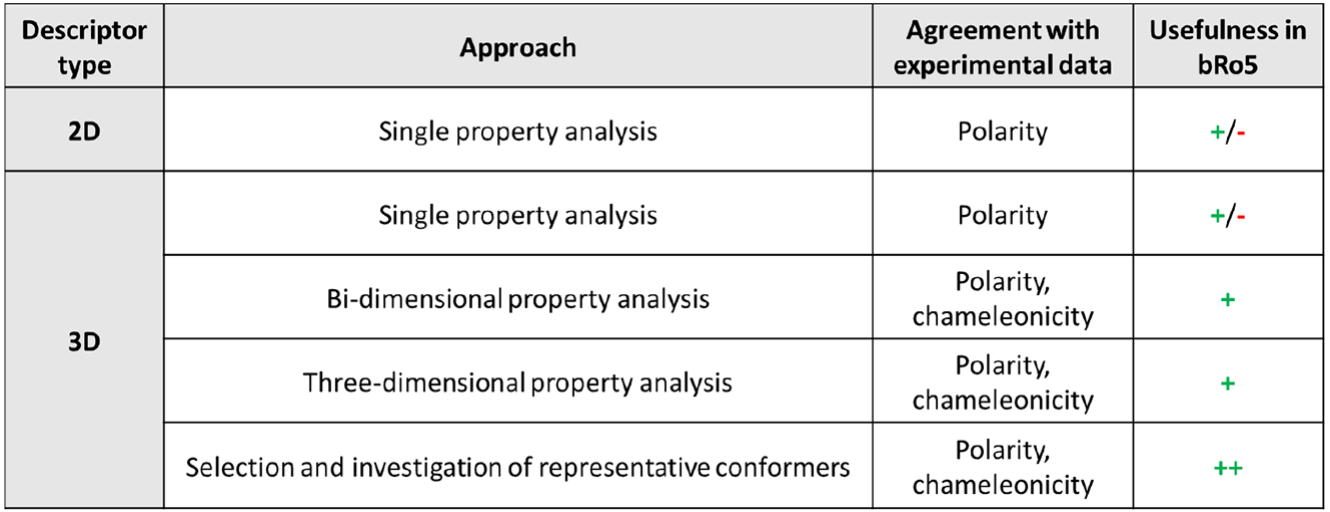
Relevance of the Applied Computational Tools for bRo5 Molecules Expressed as Agreement with Experimental Data and Usefulness in the Explored bRo5 Chemical Space

## Conclusion

A rational control of molecular properties is a key step to discover new drugs in the bRo5 chemical space. This paper considers three model compounds (one Ro5 compliant, Pomalidomide, one bRo5 drug, Saquinavir, and one PROTAC, CMP98) and highlights their different physicochemical profile using experimental and computational descriptors. A major point is to discuss whether the information content of a pool of experimental physicochemical data may be extracted from a set of pure computational analyses. Overall, the computational part provides first insights on the different behavior of the three model compounds, supporting a certain degree of chameleonicity exhibited by Saquinavir. Notably, the relation between chameleonicity and permeability remains to be clearly described and an extension of the study to more bRo5 and PROTAC compounds, with particular focus on the role of IMHBs, is in due course in our laboratories.

Nevertheless, from the methodological point of view this work achieves some important milestones: we clarified which TPSA/3D PSA method pairs can be directly confronted, and we assessed the role of Rgyr as intermolecule size and intraensemble shape descriptor. Moreover, we showed the method dependence of conformational ensembles generation. In this context, we underlined the difference between classical CS and MD-based methods by discussing that they should be compared for the final information content they provide, rather than for selected single structures. We also validated SMD tunneling to efficiently capture property changes in water and toluene by showing increased property variations within shorter simulation courses than MD. Indeed, while comparing MD to SMD tunneling, the exploration of lower Rgyr, 3D PSA, and more IMHBs suggests a role as powerful tool for challenging the capacity of molecules to behave as smaller and less polar ones, an essential feature for exploring the far bRo5 chemical space (e.g., with PROTACs).

Finally, we investigated the use of selected tridimensional descriptors to characterize the generated conformers: 3D PSA, Rgyr, and IMHB. Through combinations of them and careful selection of representative conformers, we managed to monitor the simultaneous evolution of polarity, size, shape, and intramolecular interactions for the three molecules in two solvent systems representative for biological environments, making a further step to understand the factors affecting their pharmacokinetics.

The selected three model compounds have very different chemical structures. A legitimate question is whether the applied strategies are sensitive enough to discriminate compounds inside, for instance, a series of PROTACs. According to our experience, we believe that if two compounds have a very different physicochemical profile, this difference can be caught by our approach. We still remain cautious about absolute quantifications of physicochemical properties of chameleonic molecules with a single computational method, thus at this early stage we suggest analyzing pairs rather than larger groups of compounds with different methods, suited for unraveling different aspects. Another relevant question related to the selected compounds is about ionization, which is expected to complicate the scenario. In particular, whereas we could envisage to use methods developed for neutral compounds for compounds like Saquinavir with a limited number of ionized species, with eventually simple adjustments. In any case, fully ionized compounds are expected to require in most cases ad hoc strategies and work along these lines is in due course in our laboratories.

Overall, this work represents a further step to close the gap between experimental and computational methods for bRo5 property determination, allowing a presynthesis screening. Indeed, the chosen model compounds can be computationally distinguished by (a) number of congruent conformers, (b) definition of specific property variation window, and (c) impact of IMHBs on molecular architecture. Nevertheless, we remain cautious about drawing further speculations solely based on computational data and underline that at this stage there is still the need for further experimental information before shifting to an exclusive use of computational tools.

## Materials and Methods

### Materials

CMP 98 was purchased from Tocris Bioscience and Pomalidomide and Saquinavir mesylate from Sigma-Aldrich. HPLC grade acetonitrile (ACN) and methanol were procured from VWR chemicals, and ammonium acetate from Alfa Aesar (all reagents are analytical grade). Potassium phosphate monobasic (KH_2_PO_4_) and dipotassium phosphate (K_2_HPO_4_) were bought from Carlo Erba Reagents, ACS grade. Moreover, milli-Q water was used.

### Instruments

Chromatographic measurements were performed using instrument DIONEX Ultimate 3000, Thermo Scientific Inc. coupled to RS Diode Array and Chromeleon 7.2.10 software (www.thermofisher.com) (HPLC). HPLC columns IAM.PC.DD2 (300 Å, 10 μm, 10 cm × 4.6 mm) from REGIS, XBridge Shield RP18 (130 Å, 5 μm, 5 cm × 4.6 mm) from Waters (www.waters.com) and PLRP-S polymeric reversed phase column (100 Å, 5 μm, 50 mm × 4.6 mm) from Agilent were used. Ergonomic high-performance single-channel variable volume pipettors, HPLC 1.5 mL vials, 0.1 mL microinsert, and PP screw 9 mm caps were purchased from VWR Signature. pH was controlled with Eutech pH Meter 2700 (www.fishersci.com).

### Chromatographic Methods

Mobile phases consisted of an isocratic solution of 20 mM ammonium acetate at pH 7.0 and acetonitrile at various percentages (see specific method). Samples were dissolved in buffer/acetonitrile at concentrations ranging from 50 to 100 μg/mL. Chromatographic measurements were analyzed in duplicate. Then 10 μL of each solution (volume of injection) were injected at an isocratic 1 mL/min flow rate, analyzed at 30 °C (oven temperature). Each chromatographic descriptor required a specific HPLC method (see below).

### BRlogD

The three compounds previously dissolved in ACN were injected into the X-Bridge column (mobile phase: 40% buffer, 60% ACN), and the retention times and dead time (t0, baseline disturbance) were recorded. Consequently, capacity factor log *k*′60 was calculated (log *k*′60 = [*t*_R_ – *t*_0_]/*t*_0_). The corresponding BRlogD value was obtained using equation: BRlogD = 3.31 × log *k*′60 + 2.79.^12^ The used standard is acetone, caffeine, ibuprophen, lidocaine, phenol and a mixture of uracile, acetophenone and toluene.

### log *k*_W_^IAM^

Dissolved samples were injected into column IAM.PC.DD2, and retention times were recorded at different mobile phase percentages (from 10 to 50% ACN). Capacity factor was calculated for each mobile phase condition using equation: *k*′ = [*tR* – *t*0]/*t*0) *t*0 being the retention time of a nonretained molecule (citric acid). The log *k*_W_^IAM^ value for each molecule was calculated by extrapolating the 100% buffer value (0% ACN) from the equation obtained with the five mobile phase conditions, previously mentioned. In addition, five standards (caffeine, carbamazepine, ketoprofen, theobromine, and toluene) were examined on a daily basis.^33^

### Δlog *k*_W_^IAM^

Δlog *k*_W_^IAM^ was earlier defined by Grumetto et al.^48^ as (Δlog *k*_W_^IAM^ = experimental log *k*_W_^IAM^ – clog *k*_W_^IAM^ (log *k*_W_^IAM^ for neutral and nonpolar compounds with PSA = 0). Moreover, clog *k*_W_^IAM^ was correlated to log *P* (octanol/water)^48^ and more recently to BRlogD^12^ with equation (clog *k*_W_^IAM^ = BRlogD* 0.92–1.03). Therefore, the measurement of BRlogD and log *k*_W_^IAM^ for the three samples allowed to calculate polarity descriptor Δlog *k*_W_^IAM^.

### log *k*′80 PLRP-S

The retention times of the three samples were recorded at 80% ACN and capacity factors were calculated. Moreover, gold standards (acetone, caffeine, phenol, uracil–acetophenone--toluene mix and benzene) were daily checked.^15^

### EPSA

EPSA was determined following the SFC protocol by Goetz and co-workers.^17^ Briefly, a polar stationary phase (Chirex 3014) and a nonpolar mobile phase (supercritical CO_2_ with the addition of 20 mM ammonium formate in methanol as a modifier) were used to enable separation of compounds based on their polarity. The modifier was varied in 11 min from 5% to 60% at 5%/min in a linear gradient, holding at 60% for 4.9 min and reverting to the original 5% in 0.1 min. The flow rate was 5 mL/min, with the outlet back pressure set to 100 bar instead of 140 bar of the original method. Samples were dissolved in DMSO, the injection volume was 5 μL. The column temperature was set to 40 °C.

### log *P*_tol_

log *P*_tol_ was determined with an automated, miniaturized shake flask method in a 96-well format according to the protocol described by Shalaeva and co-workers.^24^

### 2D Descriptors and Molecules

the SMILES codes of the three molecules were downloaded from Chemspider (www.chemspider.com). Pomalidomide is racemic; we considered the structure of *S*-Pomalidomide in our simulations. The 2D molecular descriptors were calculated with the following software: Dragon (https://chm.kode-solutions.net/pf/dragon-7-0/, version 7.0.10, 2017, Kode srl) and AlvaDesc (Alvascience, Software, www.alvascience.com/alvadesc/, version 1.0.18,n 2020) were used to calculate all 2D physicochemical descriptors, except the number of aromatic rings count which was verified with OSIRIS DataWarrior, http://www.openmolecules.org/datawarrior/, version 5.2.1, 2021).

**Initial 3D geometries** were generated from SMILES codes with CORINA demo (www.mnam.com/online-demos/corina_demo). For Pomalidomide, the *S* enantiomer was considered.

**Conformational Sampling**: The starting points were the stuctures generated with CORINA demo. The CS tool employed was the one implemented in the force field based molecular modeling Maestro suite (Schrödinger, release 2021-3; Maestro, version 12.3, Maestro LLC, New York, NY, 2021, and Schrödinger release 2021-3, Macromode, LLC, New York, NY, 2021). For this purpose, the force field OPLS_2005 (with default parameters) was employed.

**Molecular Dynamics**: The simulation was set up using the online input generator CHARMM-GUI (www.charmm-gui.org/). Each 3D structure geometry was first converted from.mol2 to.PDB file, and the relative CHARMM36 parameters were generated with the “Ligand reader and modeler” functionality of CHARMM-GUI. Periodic boundaries, water solvation box, and the MD input files for an NPT ensemble at 300 K were generated through the “solution builder” functionality of CHARMM-GUI.

For the toluene solution, first, a toluene molecule was constructed from scratches in VMD, then parametrized with CHARMM-GUI for the charmm36 force field, and the consequent parameters were joined to the previously generated ones specific for the desired solute. The “multicomponent assembler” of CHARMM-GUI was then employed for specifying the composition of the solution (based on toluene density at RT, 870 g/L), and a 300 K, NPT ensemble simulation folder system, comprehensive of parameters and input files was generated. NAMD2 2.13^49^ CUDA-accelerated version (www.ks.uiuc.edu/Research/namd/) was used to equilibrate the system (250 ps) and to run the production (10 ns) on a Linux workstation (OS, CentOS7, 32GB DDR2; CPU, Xeon Octa-core 3.50 GHz, Titan XP GPU).

The resulting production trajectories were visualized and cleaned off the solvent molecules with VMD^50^ (http://www.ks.uiuc.edu/Research/vmd/).

### SMD Tunneling

The same steps as for MD were followed up to the production phase. Then, the input file was accordingly modified by introduction of the following terms and parameters: SMD = on, SMDk = 7.0 kcal/mol/Å, SMDvel = 2*e*–05 Å/ts, SMDdir = 0.0, 1.0, 0.0).

The resulting coordinate file from the previous equilibration was modified in the PDB field “occupancy”, in order to allow the recognition of the solute molecules as target for the SMD additional velocity term.

### IMHBs Determination

The formation of intramolecular hydrogen bonds (IMHBs) was explored with USCF Chimera 1.15 (https://www.rbvi.ucsf.edu/chimera/). Default chemical requirements were used (hydrogens bound to nitrogen, oxygen, and sulfur as donors and nitrogen, oxygen, and sulfur atoms with lone pairs as acceptors), whereas a relaxation of 0.4 Å (bond distance) and 20° (angle between the HBD-HBA) was accepted.^46^

### 3D Descriptors

The calculation of 3D PSA, Rgyr, and log *P*(MLP) was performed in VEGA ZZ^44^ (http://www.vegazz.net/) by import of either a.mol2 file (conformers from CS and the crystallographic structures of Saquinavir), or a.trr file resulting from MD and SMD tunneling after removal of the solvent molecules. All descriptors calulated in Vega ZZ had standard settings (essential, probe radius of 3D PSA was 0).

### Data Analysis, Graphical Analysis, and Rendering

Data analysis was performed with Microsoft Excel version 2010 (www.microsoft.com) and finalized by importing the data in OSIRIS DataWarrior (http://www.openmolecules.org/datawarrior/, version 5.2.1, 2021) or GraphPad Prism, version 9.0 (www.graphpad.com).

The color-coded density plots were performed with the r package ggplot2 (https://cran.r-project.org/web/packages/ggplot2). The same logic of a Ramachandran’s angle density plotting was applied:^51^ the 2D plot was divided in tiles, and for each conformer for which properties fall within the tile, a score is assigned. The tiles are then color-coded according to the density score and reveal where most conformers fall in the 2D property plot.

Visual inspection and extraction of the conformer images was performed with Vega ZZ^44^ (http://www.vegazz.net/).

## Supporting Information Available

Parameters for IMHB detection; Pomalidomide. 2D plot of 3D PSA vs Rgyr; comparison between short SMD tunneling and longer MD simulations; density plots; average IMHB number within clusters from density plots; HPLC traces; solubility and permeability data; calculated log *P* data; energy window of the system; number of conformer generated with CS; original TPSA training data set; rigid compounds and calculation of TPSA/3D PSA; Pearson’s correlation matrix of PSA methods; curve slopes for PSA pairs correlation; *Y* intercepts for PSA pairs correlation; comparable PSA/TPSA pairs; relevant angles and distances for IMHB detection (PDF)
Molecular formula strings (CSV)

## Abbreviations

3D PSA: tridimensional polar surface area
ADME: adsorption distribution metabolism escretion
bRo5: beyond the rule of 5
CS: conformational sampling
IAM: immobilized artificial membranes
IMHB: intramolecular hydrogen bond
MD: molecular dynamics
POI: protein of interest
PROTAC: proteolysis targeting chimera
Rgyr: radius of gyration
Ro5: rule of 5
SMD: steered molecular dynamics
TPD: targeted protein degradation
TPSA: topological polar surface area
