# Supplementary material for: Refinement of Computational Access to Molecular Physicochemical Properties: From Ro5 to bRo5
Source: J Med Chem. 2022 Sep 12;65(18):12068–83. doi: 10.1021/acs.jmedchem.2c00774 (PMC9511483; doi:10.1021/acs.jmedchem.2c00774)
Supplement: Supplementary file 1 — jm2c00774_si_001.pdf [file jm2c00774_si_001.pdf]

Supporting information

Refinement of computational access to molecular physicochemical properties: from Ro5 to bRo5

*Matteo Rossi Sebastiano**,†, *Diego Garcia Jimenez*†, *Maura Vallaro*†, *Giulia Caron*†, *Giuseppe Ermondi**,†

†University of Torino, Molecular Biotechnology and Health Sciences Dept., CASSMedChem, via Quarello 15, 10135 Torino, Italy.

Corresponding Authors' email addresses: giuseppe.ermondi@unito.it, matteo.rossisebastiano@unito.it

List of contents

Supplementary File

Supporting information for publication.csv, contains formula strings.

Supplementary Methods

Definition of comparable TPSA/3D PSA pairs (Page S4)

Supplementary Figures

Figure S1: Relevant angles and distances for IMHB detection in pomalidomide (Page S5).

Figure S2: Pomalidomide. 2D plot of 3D PSA vs Rgyr (Page S6).

Figure S3: Comparison between short SMD tunneling and longer MD simulations (Page S7).

Figure S4: Density plots of saquinavir and CMP 98 in water and toluene from MD (Page S8).

Figure S5: Density plots (Fig 7), water and toluene conformers pooled together (Page S9).

Figure S6: Distribution of IMHB regions based on size and polarity of MD conformers (Page S10).

Figure S7: Conformer selection upon closeness to the center of the MD density plot (Page S11).

Figure S8: Average IMHB number within the inner clusters individuated with the the density plots in figure 11 (Page S12).

Figure S9: HPLC traces (Page S13).

Supplementary Tables

Table S1: Solubility and permeability data of the three selected compounds (Page S14).

Table S2: Calculated logP data of the three selected compounds (Page S15).

Table S3: Energy window of the system (kcal/mol) used to generate the conformers (Page S16).

Table S4: Number of conformer generated by CS with Schrödinger (Page S17).

Table S5: Original dataset used for TPSA training (Pages S18-S34).

Table S6: selection of 17 rigid compounds and calculation of TPSA and 3D PSA methods (Pages S35-S38).

Table S7: Pearson's correlation matrix for TPSA and 3D PSA methods (Pages S39-S40).

Table S8: Curve slopes for TPSA and 3D PSA correlations (Page S41).

Table S9: Y Intercepts for TPSA and 3D PSA correlations (Page S42).

Table S10: Comparable PSA/TPSA pairs (Page S43).

Table S11: Relevant angles and distances for IMHB detection with sp2-O acceptors (HBA, Page S44).

Table S12: Relative 3D PSA differences in the generated conformers ensembles (Page S45).

Table S13: Relative Rgyr differences in the generated conformers ensembles (Page S46).

Supplementary methods: TPSA/3D PSA pairs definition

We decided to take the original dataset by Clark and colleagues and their 3D PSA published values,[41] upon which Ertl and co-workers[40] developed their TPSA definitions. Among the 102 molecules composing the dataset we selected the most rigid ones with either 0 or 1 rotatable bonds (Supplementary, Table S5). We then calculated TPSA values with 5 different methods and generated 3D geometries for those molecules with CORINA demo (www.molecular-networks.com). Given the reduced size and high rigidity of these Ro5 molecules, we are confident that the approximation to one single 3D conformer is representative enough for 3D PSA calculation. We then calculated 3D PSA values with 5 different methods and correlated them with the originally published 3D PSA[41] and the different TPSA methods (Supplementary, Table S6). Given the information provided by some of the TPSA, 3D PSA methods, the correlation coefficients (Supplementary Table, S7), the slopes (Supplementary Table S8) and intercept (Supplementary Table S9) of the fitted curves, we were able to estimate which atom selections are considered by each method and which TPSA/3D PSA methods are comparable (Supplementary Table S10).

Figure S1: Relevant angles and distances for IMHB formation with sp2-O acceptors (HAcc) in Pomalidomide. The IMHB is recognized just when the relaxation threshold is extended over 20°.

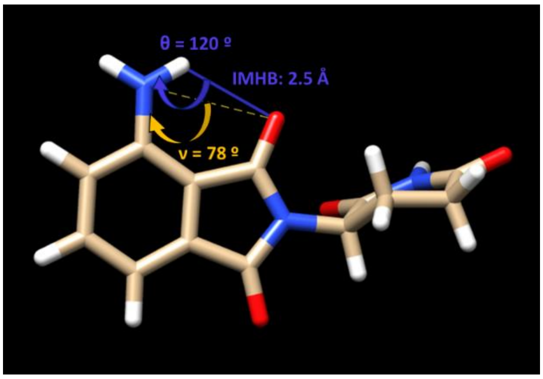

Figure S2: Pomalidomide. 2D plot of 3D PSA vs Rgyr for (A) CS in water and chloroform, (B) MD (water, toluene) and (C) SMD tunneling (water, toluene).

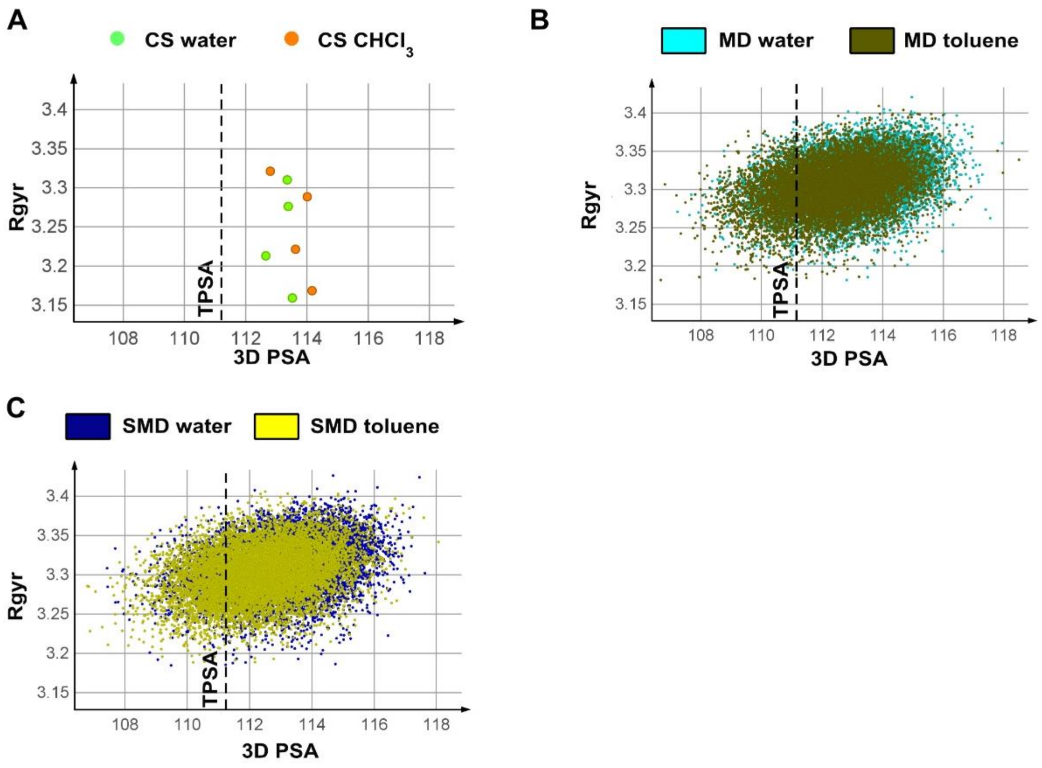

Figure S3: comparison of SMD tunneling (10ns) and 10 folds longer MD simulation evaluated with the bi-dimensional analysis. (A) saquinavir, (B) CMP 98.

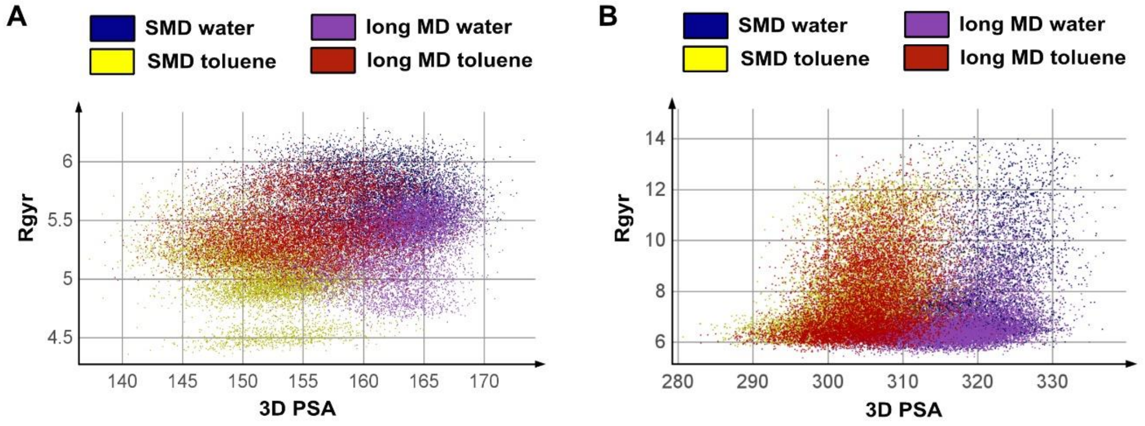

Figure S4: Density plots of saquinavir in water (A) and toluene (B) and CMP 98 in water (C) and tolu-ene (D) from MD highlighting the dispersion patterns of the generated conformers in the 2D plot of 3D PSA vs Rgyr. The color scale is expressed as conformer frequency per tile. Blue perimeter stands for water, orange for toluene. Orange/blue crosses highlight the solvent-based shift of the inner cluster.

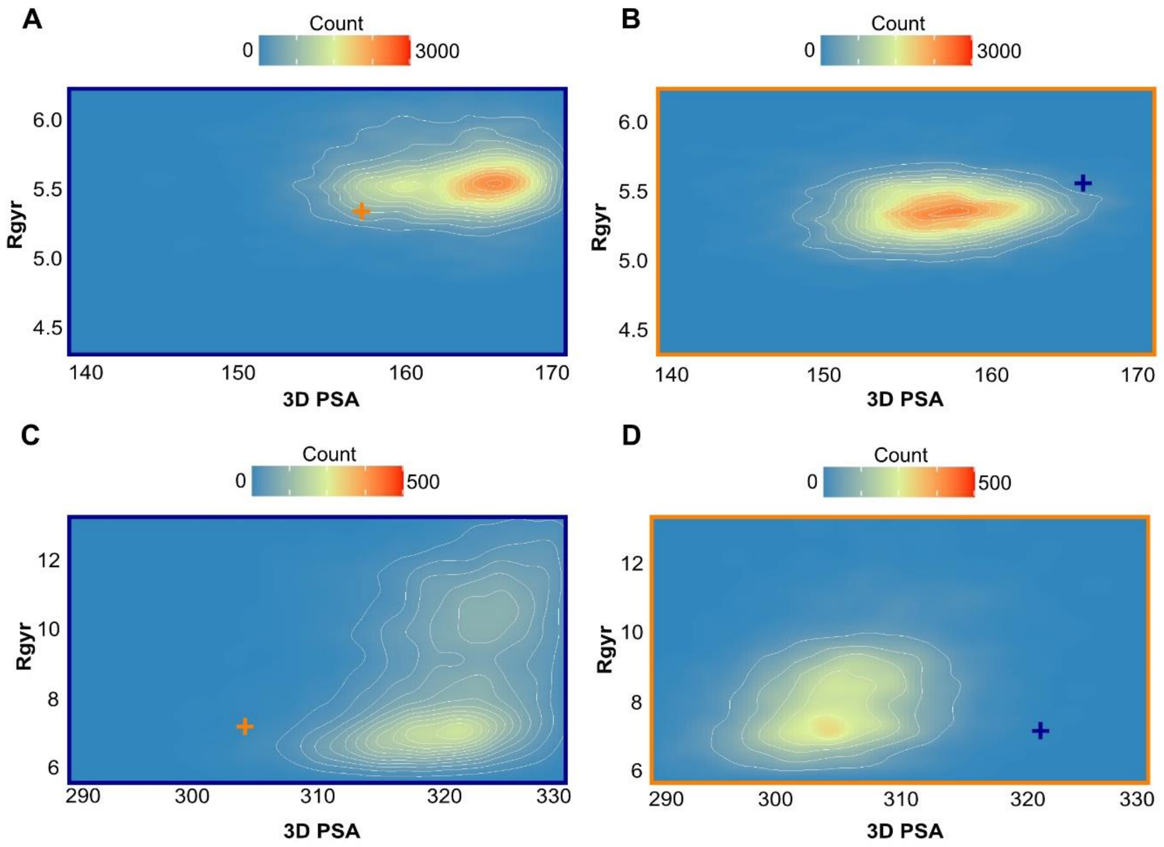

Figure S5: Density plots of Saquinavir (A) and CMP 98 (B) of the SMD conformers generated in water and toluene (pooled together) when plotting 3D PSA vs Rgyr in 2D. The density is expressed as count per tile.

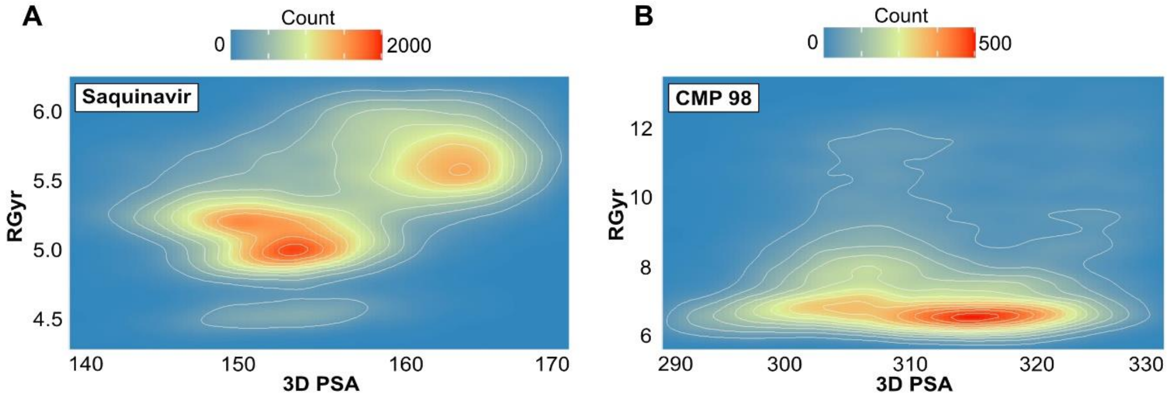

Figure S6: Distribution of IMHB regions based on size and polarity of MD conformers. A, B) Saquinavir in water (A) and toluene (B). C, D) CMP 98 in water (C) and toluene (D). Blue perimeter stands for water, orange for toluene.

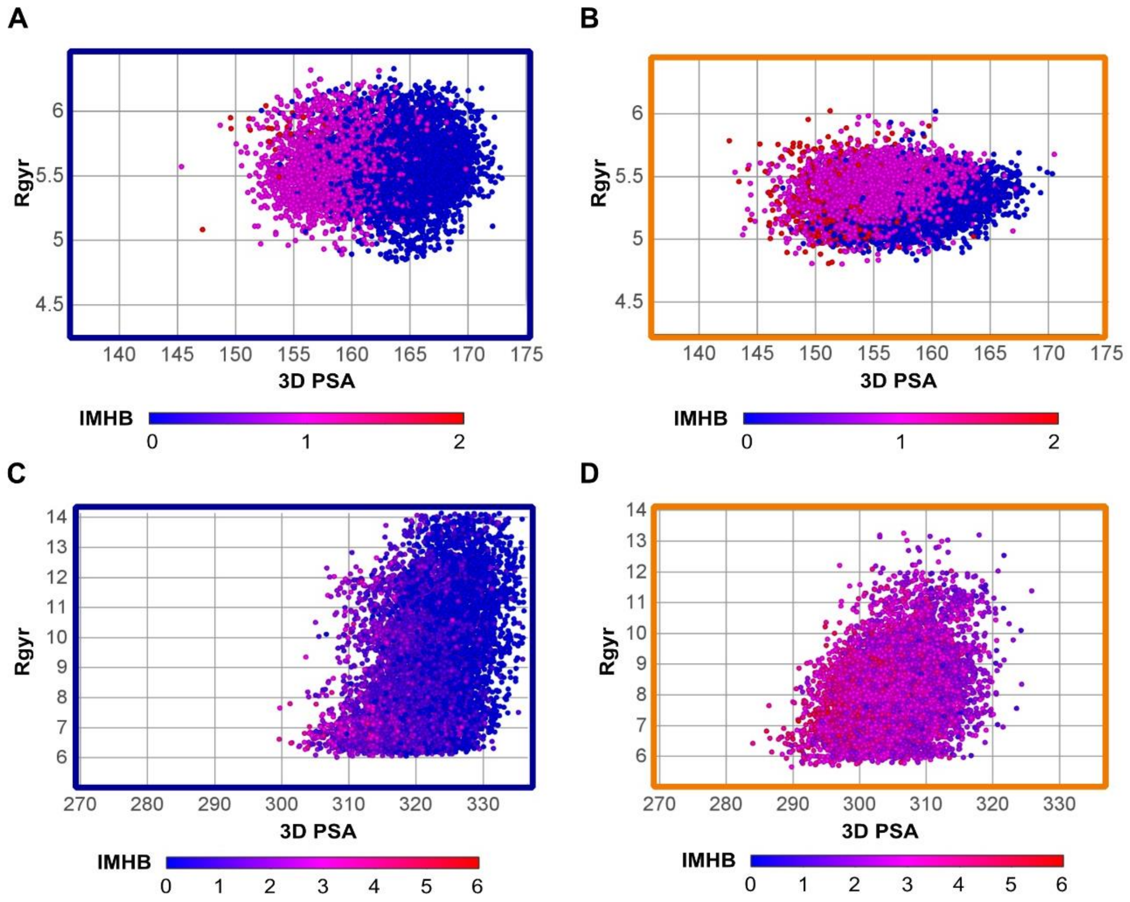

Figure S7: Conformer selection upon closeness to the center of the MD density plot (Figure S6). Blue perimeter stands for water, orange for toluene. IMHB are depicted by dashed ovals and lines, the missing IMHB of CMP 98 with the lighter dashed oval. A) Saquinavir (purple) MD conformer. B) CMP 98 (green) MD conformer.

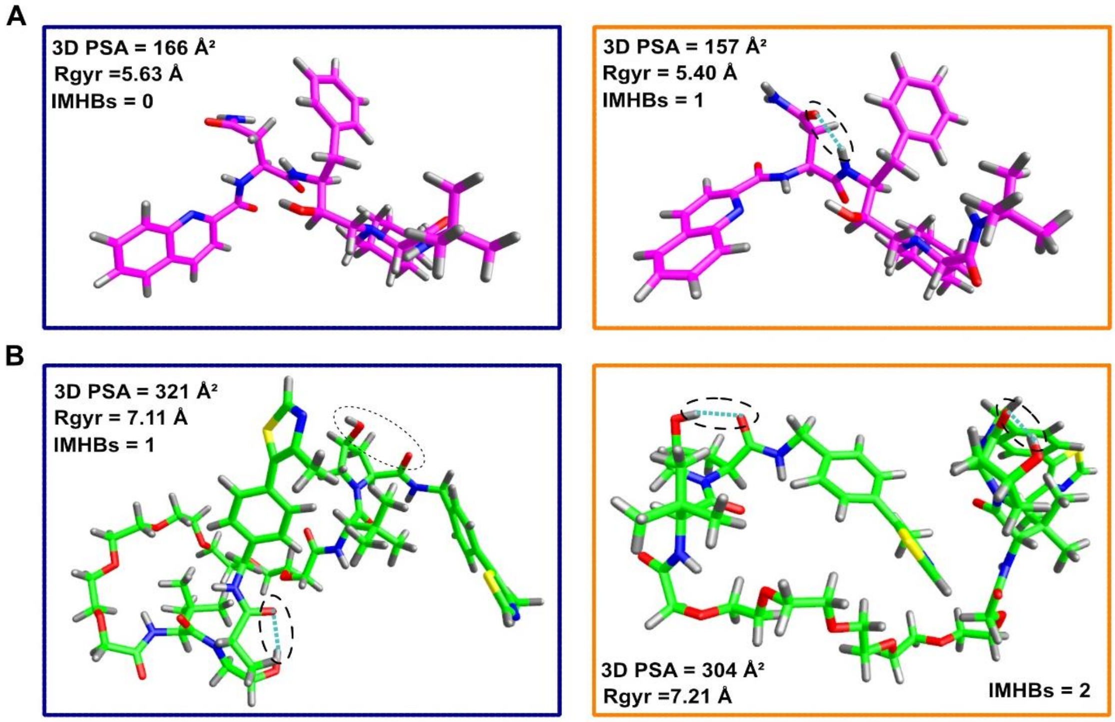

Figure S8: Average IMHB number within the inner clusters of the density plots of SMD conformers.

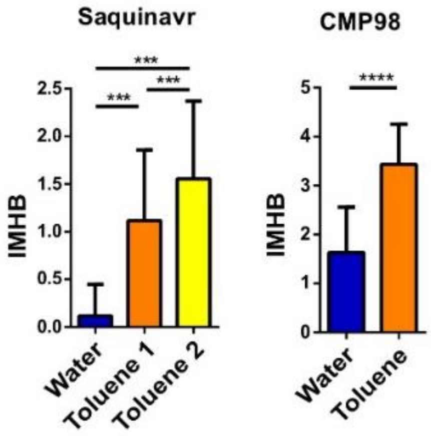

Figure S9: HPLC traces (IAM column)

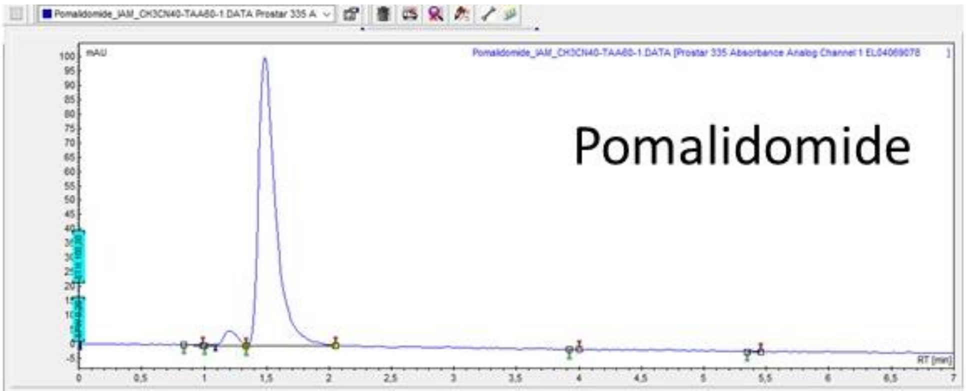

#	Name	Time [Min]	Quantity [% Area]	Height [mAU]	Area [mAU.Min]	Area % [%]	Lipophilicity index
1	UNKNOWN	0,91	0,07	0,2	0,0	0,075	0,70
2	UNKNOWN	1,07	0,12	0,4	0,0	0,118	1,00
3	UNKNOWN	1,20	4,01	5,2	0,7	4,013	1,25
4	UNKNOWN	1,48	95,63	100,3	15,9	95,628	1,77
5	UNKNOWN	3,96	0,03	0,2	0,0	0,034	6,42
6	UNKNOWN	5,39	0,13	0,4	0,0	0,133	9,09
Total			100,00	106,7	15,7	100,000	20,22

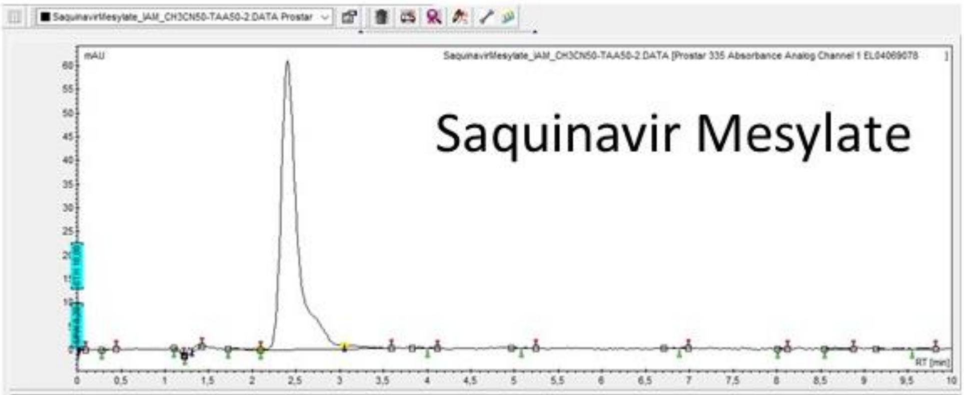

#	Name	Time [Min]	Quantity [% Area]	Height [mAU]	Area [mAU.Min]	Area % [%]	Lipophilicity index
1	UNKNOWN	0,05	0,07	0,2	0,0	0,071	-0,90
2	UNKNOWN	0,36	0,04	0,0	0,0	0,037	-0,33
3	UNKNOWN	1,16	0,33	0,7	0,0	0,326	1,17
4	UNKNOWN	1,27	0,47	1,3	0,1	0,466	1,37
5	UNKNOWN	1,36	0,47	0,7	0,1	0,472	1,55
6	UNKNOWN	2,01	0,29	0,3	0,0	0,295	2,77
7	UNKNOWN	2,40	96,28	60,9	13,2	96,285	3,49
8	UNKNOWN	3,11	1,26	0,6	0,2	1,261	4,82
9	UNKNOWN	4,05	0,09	0,3	0,0	0,095	6,59
10	UNKNOWN	5,12	0,10	0,2	0,0	0,103	8,59
11	UNKNOWN	6,93	0,09	0,2	0,0	0,091	11,98
12	UNKNOWN	8,05	0,05	0,1	0,0	0,051	14,08
13	UNKNOWN	8,68	0,22	0,3	0,0	0,224	15,25
14	UNKNOWN	9,68	0,23	0,1	0,0	0,226	17,13
Total			100,00	66,0	13,7	100,000	87,57

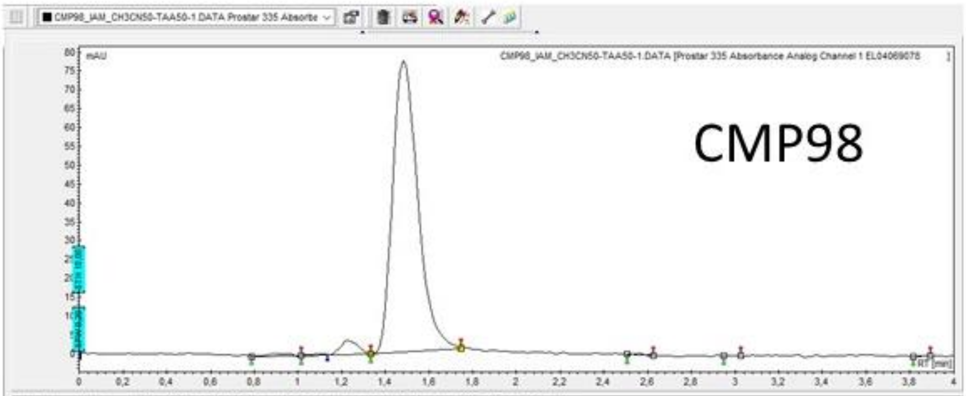

#	Name	Time [Min]	Quantity [% Area]	Height [mAU]	Area [mAU.Min]	Area % [%]	Lipophilicity index
1	UNKNOWN	0,95	0,62	0,5	0,1	0,623	0,77
2	UNKNOWN	1,08	0,27	0,4	0,0	0,269	1,02
3	UNKNOWN	1,23	3,22	3,8	0,3	3,216	1,30
4	UNKNOWN	1,48	95,67	77,2	10,3	95,666	1,77
5	UNKNOWN	2,56	0,13	0,4	0,0	0,130	3,79
6	UNKNOWN	2,97	0,05	0,1	0,0	0,045	4,57
7	UNKNOWN	3,05	0,05	0,2	0,0	0,052	6,22
Total			100,00	82,5	10,8	100,000	19,44

Table S1: Thermodynamic solubility and permeability data of the three selected compounds.

Molecular descriptors & Molecules	Solubility 25ºC log S (mol/l)	Permeability (cm/s x 10^{-6})
**Pomalidomide**	-4.55	24.9 (Caco-2)[13]
**Saquinavir**	NM[52]	Mainly active transport (Caco-2 with efflux inhibiton: 5.0)[11]
**CMP 98**	-3.48	0.0 (PAMPA)[14]

Table S2: Calculated log values for the three selected compounds with three different methods: atom-based (chemaxon), property-based (MlogP) and fragment-based (miLogP) strategies.[53]

	Calculated lipophilicity		
	AlogP-based (Marvin; chemaxon)	MlogP (Swissadme)	MiLogP (Molinspiration)
Pomalidomide	-0.49	0.74	-0.54
Saquinavir	2.51	1.4	4.26
CMP 98	-0.7	-3.47	1.67

Table S3: Energy window of the system (kcal/mol) used to generate the conformers.

	CS		MD		SMD	
**Molecule**	**$CHCl_3$**	**Water**	**Toluene**	**Water**	**Toluene**	**Water**
Pomalidomide	0.063513	0.298547	633.9579	705.4989	715.3257	593.1015
Saquinavir	4.949582	5.00554	465.0536	565.8814	462.9815	547.8239
CMP 98	4.986041	5.00301	943.1956	1148.892	1229.518	1168.96

Table S4: Conformers number generated with Schroedinger. Data bars in green are calculated on all fields.

Molecule	Tot conformers	Water conformers	Chloroform conformers
Pomalidomide	8	4	4
Saquinavir	164	126	38
CMP 98	189	73	116

Table S5: Original dataset of small molecules used by Clark and colleagues[41] to calculate 3D PSA upon which Ertl and co-workers developed the TPSA.[40] Different 2D descriptors are reported. Of note RBN (number of rotable blonds) constituted the rigidity selection criteria.

Compound	SMILES	MW	RBN	PHI	TPSA(NO) - Dragon	TPSA(Tot) - Dragon
Caffeine	CN1C=NC2=C1C(=O)N(C)C(=O)N2C	194.22	**0**	1.855	61.82	61.82
Coumarin	O=C1OC2=CC=CC=C2C=C1	146.15	**0**	1.337	30.21	30.21
Testosterone	C[C@]12CC[C@H]3[C@@H](CCC4=CC(=O)CC[C@]34C)[C@@H]1CC[C@@H]2O	288.47	**0**	2.962	37.3	37.3
Theophylline	CN1C2=C(NC=N2)C(=O)N(C)C1=O	180.19	**0**	1.631	72.68	72.68
Acetaminophen	CC(=O)NC1=CC=C(O)C=C1	151.18	**1**	2.362	49.33	49.33
Bromazepam	BrC1=CC=C2NC(=O)CN=C(C3=CC=CC=N3)C2=C1	316.17	**1**	3.24	54.35	54.35

Chlorothiazide	NS(=O)(=O)C1=CC2=C(NC=NS2(=O)=O)C=C1Cl	295.75	**1**	2.763	118.69	135.45
Diazepam	CN1C2=CC=C(Cl)C=C2C(=NCC1=O)C1=CC=CC=C1	284.76	**1**	3.39	32.67	32.67
Foscarnet	OC(=O)P(O)(O)=O	126.01	**1**	1.67	94.83	104.64
Hydrochlorothia-zide	NS(=O)(=O)C1=CC2=C(NCNS2(=O)=O)C=C1Cl	297.77	**1**	2.937	118.36	135.12
Lamotrigine	NC1=NN=C(C(N)=N1)C1=CC=CC(Cl)=C1Cl	256.11	**1**	3.028	90.71	90.71
Lormetazepam	CN1C2=CC=C(Cl)C=C2C(=NC(O)C1=O)C1=CC=CC=C1Cl	335.2	**1**	4.019	52.9	52.9
Nordiazepam	ClC1=CC=C2NC(=O)CN=C(C3=CC=CC=C3)C2=C1	270.73	**1**	3.158	41.46	41.46
Oxazepam	OC1N=C(C2=CC=CC=C2)C2=CC(	286.73	**1**	3.365	61.69	61.69

	Cl)=CC=C2NC1=O					
Phenazone	CN1N(C(=O)C=C1C)C1=CC=CC=C1	188.25	**1**	2.196	26.93	26.93
Progesterone	CC(=O)[C@H]1CC[C@H]2[C@@H]3CCC4=CC(=O)CC[C@]4(C)[C@H]3CC[C@]12C	314.51	**1**	3.422	34.14	34.14
Salicylic Acid	OC(=O)C1=CC=CC=C1O	138.13	**1**	1.804	57.53	57.53
Clonidine	ClC1=CC=CC(Cl)=C1NC1=NCCN1	230.11	2	3.031	36.42	36.42
Corticosterone	C[C@]12C[C@H](O)[C@H]3[C@@H](CCC4=CC(=O)CC[C@]34C)[C@@H]1CC[C@@H]2C(=O)CO	346.51	2	4.013	74.6	74.6
Dexamethasone	C[C@@H]1C[C@H]2[C@@H]3CCC4=CC(=O)C=C[C@]4(C)[C@@]3(F)[C@@H](O)C[	392.51	2	4.161	94.83	94.83

	C@]2(C)[C@@]1(O)C(=O)CO					
Guanabenz	NC(N)=N\N=C\C1=C(Cl)C=CC=C1Cl	231.1	2	4.037	76.76	76.76
Hydrocortisone	C[C@]12C[C@H](O)[C@H]3[C@@H](CCC4=CC(=O)CC[C@]34C)[C@@H]1CC[C@]2(O)C(=O)CO	362.51	2	4.046	94.83	94.83
Methylpredniso-lone	C[C@H]1C[C@H]2[C@@H]3CC[C@](O)(C(=O)CO)[C@@]3(C)C[C@H](O)[C@@H]2[C@@]2(C)C=CC(=O)C=C12	374.52	2	4.132	94.83	94.83
Metolazone	CC1NC2=CC(Cl)=C(C=C2C(=O)N1C1=CC=CC=C1C)S(N)(=O)=O	365.87	2	4.238	92.5	100.88
Ondansetron	CN1C2=C(C3=CC=CC=C13)C(=O)C(CN1C=CN=C1C)CC2	293.4	2	3.103	39.82	39.82

Phenytoin	O=C1NC(=O)C(N1)(C1=CC=CC=C1)C1=CC=CC=C1	252.29	2	2.748	58.2	58.2
Prednisolone	C[C@]12C[C@H](O)[C@H]3[C@@H](CCC4=CC(=O)C=C[C@]34C)[C@@H]1CC[C@]2(O)C(=O)CO	360.49	2	3.902	94.83	94.83
Propylthiouracil	CCCC1=CC(=O)NC(=S)N1	170.26	2	2.879	48.65	80.74
Tenidap	NC(=O)N1C(O)=C(C(=O)C2=CC=CS2)C2=CC(Cl)=CC=C12	320.77	2	3.569	85.32	113.56
Tranexamic Acid	NC[C@H]1CC[C@@H](CC1)C(O)=O	157.24	2	3.032	63.32	63.32
Acetylsalicylic Acid	CC(=O)OC1=CC=CC=C1C(O)=O	180.17	3	2.903	63.6	63.6
Captopril	C[C@H](CS)C(=O)N1CCC[C@H]1C(O)=O	217.32	3	4.108	57.61	96.41
Ciprofloxacin	OC(=O)C1=CN(C2CC2)C2=CC(N3	331.38	3	3.816	74.57	74.57

	CCNCC3)=C(F)C=C2C1=O					
Gabapentin	NCC1(CC(O)=O)CCCCC1	171.27	3	3.238	63.32	63.32
Naproxen	COC1=CC2=CC=C(C=C2C=C1)[C@H](C)C(O)=O	230.28	3	3.227	46.53	46.53
Norfloxacin	CCN1C=C(C(O)=O)C(=O)C2=CC(F)=C(C=C12)N1CCNCC1	319.37	3	4.374	74.57	74.57
Sorivudine	OC[C@H]1O[C@H]([C@@H](O)[C@@H]1O)N1C=C(\C=C\Br)C(=O)NC1=O	349.16	3	4.822	124.78	124.78
Tiacrilast	CSC1=CC=C2N=CN(\C=C\C(O)=O)C(=O)C2=C1	262.31	3	3.723	72.19	97.49
Trovafloxacin	N[C@H]1[C@@H]2CN(C[C@H]12)C1=C(F)C=C2C(=O)C(=CN(C3=CC=C(F)C=C3F)C2=N1)C(O)=O	416.39	3	4.177	101.45	101.45

Zidovudine	CC1=CN([C@H]2C[C@H](N=[N+]=[N-])[C@@H](CO)O2)C(=O)NC1=O	267.28	3	3.955	133.08	133.08
Amoxicillin	CC1(C)S[C@@H]2[C@H](NC(=O)[C@H](N)C3=CC=C(O)C=C3)C(=O)N2[C@H]1C(O)=O	365.45	4	4.577	132.96	158.26
Bupropion	CC(NC(C)(C)C)C(=O)C1=CC=CC(Cl)=C1	239.77	4	4.113	29.1	29.1
Cephalexin	CC1=C(N2[C@H](SC1)[C@H](NC(=O)[C@H](N)C1=CC=CC=C1)C2=O)C(O)=O	347.43	4	4.673	112.73	138.03
Desipramine	CNCCCN1C2=CC=CC=C2CCC2=CC=CC=C12	266.42	4	4.145	15.27	15.27
Ibuprofen	CC(C)CC1=CC=C(C=C1)C(C)C(O)=O	206.31	4	4.043	37.3	37.3

Imipramine	CN(C)CCCN1C2=CC=CC=C2CCC2=CC=CC=C12	280.45	4	4.375	6.48	6.48
Ketoprofen	CC(C(O)=O)C1=CC=CC(=C1)C(=O)C1=CC=CC=C1	254.3	4	3.885	54.37	54.37
Loracarbef	N[C@@H](C(=O)N[C@H]1[C@H]2CCC(Cl)=C(N2C1=O)C(O)=O)C1=CC=CC=C1	349.8	4	4.629	112.73	112.73
Olsalazine	OC(=O)C1=CC(=CC=C1O)\N=N\C1=CC=C(O)C(=C1)C(O)=O	302.26	4	4.509	139.78	139.78
Prazosin	COC1=CC2=NC(=NC(N)=C2C=C1OC)N1CCN(CC1)C(=O)C1=CC=CO1	383.45	4	5.283	106.95	106.95
Quinidine	COC1=CC2=C(C=CN=C2C=C1)[C@H](O)[C@H]1C[C@@H]2CCN1C[C@@H]2C=C	324.46	4	4.13	45.59	45.59

Terazosin	COC1=CC2=NC(=NC(N)=C2C=C1OC)N1CCN(CC1)C(=O)C1CCCO1	387.49	4	5.642	103.04	103.04
Terbutaline	CC(C)(C)NCC(O)C1=CC(O)=CC(O)=C1	225.32	4	4.047	72.72	72.72
Warfarin	CC(=O)CC(C1=CC=CC=C1)C1=C(O)C2=CC=CC=C2OC1=O	308.35	4	4.199	67.51	67.51
Ziprasidone	ClC1=CC2=C(CC(=O)N2)C=C1CCN1CCN(CC1)C1=NSC2=CC=CC=C12	412.98	4	4.993	48.47	76.71
Chloramphenicol	OC[C@@H](NC(=O)C(Cl)Cl)[C@H](O)C1=CC=C(C=C1)[N+]([O-])=O	323.15	5	6.482	115.38	115.38
Doxorubicin	COC1=CC=CC2=C1C(=O)C1=C(O)C3=C(C[C@](O)(C[C@@H]3O[C@H]3C[C@H](N)[C@H](O)[C@H]	543.57	5	7.056	206.07	206.07

	(C)O3)C(=O)CO)C(O)=C1C2=O					
Etoposide	COC1=CC(=CC(OC)=C1O)[C@H]1[C@@H]2[C@H](COC2=O)[C@H](O[C@@H]2O[C@@H]3CO[C@@H](C)O[C@H]3[C@H](O)[C@H]2O)C2=CC3=C(OCO3)C=C12	588.61	5	7.428	160.83	160.83
Fluconazole	OC(CN1C=NC=N1)(CN1C=NC=N1)C1=CC=C(F)C=C1F	306.31	5	3.861	81.65	81.65
Furosemide	NS(=O)(=O)C1=CC(C(O)=O)=C(NCC2=CC=CO2)C=C1Cl	330.77	5	4.581	122.63	131.01
Ganciclovir	NC1=NC(=O)C2=C(N1)N(COC(CO)CO)C=N2	255.27	5	3.929	139.28	139.28
Lactulose	OC[C@H]1O[C@](O)(CO)[C@@H](O)[C@@H]1O[C@@H]1O[C@H](CO)[C@H](O)[	342.34	5	5.93	189.53	189.53

	C@H](O)[C@H]1O					
Mannitol	OC[C@@H](O)[C@@H](O)[C@H](O)[C@H](O)CO	182.2	5	5.313	121.38	121.38
Phe-noxymethylpeni-cillinic Acid	CC1(C)S[C@@H]2[C@H](NC(=O)COC3=CC=CC=C3)C(=O)N2[C@H]1C(O)=O	350.43	5	4.599	95.94	121.24
Scopolamine	CN1[C@H]2C[C@@H](C[C@@H]1[C@H]1O[C@@H]21)OC(=O)[C@H](CO)C1=CC=CC=C1	303.39	5	3.538	62.3	62.3
Trimethoprim	COC1=CC(CC2=CN=C(N)N=C2N)=CC(OC)=C1OC	290.36	5	4.957	105.51	105.51
Valproic Acid	CCCC(CCC)C(O)=O	144.24	5	5.226	37.3	37.3
Acrivastine	CC1=CC=C(C=C1)C(=C/CN1CCCC1)\C1=CC=CC(\C=C\C(O)=O)=N1	348.48	6	6.112	53.43	53.43

Felodipine	CCOC(=O)C1=C(C)NC(C)=C(C1C1=CC=CC(Cl)=C1Cl)C(=O)OC	384.28	6	6.548	64.63	64.63
Nadolol	CC(C)(C)NCC(O)COC1=CC=CC2=C1C[C@H](O)[C@H](O)C2	309.45	6	5.584	81.95	81.95
Pindolol	CC(C)NCC(O)COC1=C2C=CNC2=CC=C1	248.36	6	4.497	57.28	57.28
Propranolol	CC(C)NCC(O)COC1=CC=CC2=CC=CC=C12	259.38	6	4.912	41.49	41.49
Sotalol	CC(C)NCC(O)C1=CC=C(NS(C)(=O)=O)C=C1	272.41	6	5.29	78.43	86.81
Sulfasalazine	OC(=O)C1=CC(=CC=C1O)\N=N\C1=CC=C(C=C1)S(=O)(=O)NC1=CC=CC=N1	398.43	6	5.687	141.31	149.69
Sulpiride	CCN1CCCC1CNC(=O)C1=CC(=CC=C1OC)S(N)(=O)=O	341.48	6	5.895	101.73	110.11

Sumatriptan	CNS(=O)(=O)CC1=CC=C2NC=C(CCN(C)C)C2=C1	295.45	6	4.605	65.2	73.58
Cefatrizine	N[C@@H](\C(O)=N\[C@H]1[C@H]2SCC(CSC3=CN=NN3)=C(N2C1=O)C(O)=O)C1=CC=C(O)C=C1	462.56	7	6.477	178.02	228.62
Cimetidine	CN\C(NCCSCC1=C(C)NC=N1)=N\C#N	252.39	7	6.524	88.89	114.19
Diltiazem	COC1=CC=C(C=C1)[C@@H]1SC2=CC=CC=C2N(CCN(C)C)C(=O)[C@@H]1OC(C)=O	414.57	7	7.224	59.08	84.38
Gentamicin	CNC(C)C1CCC(N)C(OC2C(N)CC(N)C(OC3OCC(C)(O)C(NC)C3O)C2O)O1	477.69	7	9.099	199.73	199.73
Practolol	CC(C)NCC(O)COC1=CC=C(NC(C)=O)C=C1	266.38	7	6.668	70.59	70.59

Timolol Maleate	CC(C)(C)NC[C@H](O)COC1=NSN=C1N1CCOCC1	316.48	7	6.164	79.74	107.98
Alprenolol	CC(C)NCC(O)COC1=CC=CC=C1CC=C	249.39	8	6.653	41.49	41.49
Atenolol	CC(C)NCC(O)COC1=CC=C(CC(N)=O)C=C1	266.38	8	6.668	84.58	84.58
Bumetanide	CCCCNC1=CC(=CC(=C1OC1=CC=CC=C1)S(N)(=O)=O)C(O)=O	364.46	8	6.421	118.72	127.1
Ceftriaxone	CO\N=C(/C(=O)N[C@H]1[C@H]2SCC(CSC3=NC(=O)C(=O)NN3C)=C(N2C1=O)C(O)=O)C1=CSC(N)=N1	554.65	8	8.133	214.96	293.8
Cefuroxime	CO\N=C(\C(=O)N[C@H]1[C@H]2SCC(COC(N)=O)=C(N2C1=O)C(O)=O)C1=CC=CO1	424.43	8	6.638	173.76	199.06
Cromolyn	OC(COC1=CC=C2OC(=CC(=O)C2=C1)C(O)=O)CO	468.39	8	6.697	173.71	173.71

	C1=C2C(=O)C=C(OC2=CC=C1)C(O)=O					
Enalaprilat	C[C@H](N[C@@H](CCC1=CC=CC=C1)C(O)=O)C(=O)N1CCC[C@H]1C(O)=O	348.44	8	6.97	106.94	106.94
Fluvastatin	CC(C)N1C(\C=C\[C@H](O)C[C@H](O)CC(O)=O)=C(C2=CC=CC=C12)C1=CC=C(F)C=C1	411.51	8	6.959	82.69	82.69
Labetalol	CC(CCC1=CC=CC=C1)NCC(O)C1=CC=C(O)C(=C1)C(N)=O	328.45	8	6.644	95.58	95.58
Raffinose	OC[C@H]1O[C@@](CO)(O[C@H]2O[C@H](CO[C@H]3O[C@H](CO)[C@H](O)[C@H](O)[C@H]3O)[C@@H](O)[C@H](O)[C@H]2O)[C@@H](O)[C@@H]1O	504.5	8	9.199	268.68	268.68

Methotrexate	CN(CC1=NC2=C(N)N=C(N)N=C2N=C1)C1=CC=C(C=C1)C(=O)N[C@@H](CCC(O)=O)C(O)=O	454.5	9	7.582	210.54	210.54
Metoprolol	COCCC1=CC=C(OCC(O)CNC(C)C)C=C1	267.41	9	7.675	50.72	50.72
Oxprenolol	CC(C)NCC(O)COC1=CC=CC=C1OCC=C	265.39	9	7.371	50.72	50.72
Acebutolol	CCCC(=O)NC1=CC=C(OCC(O)CNC(C)C)C(=C1)C(C)=O	336.48	10	8.794	87.66	87.66
Ranitidine	CN\C(NCCSCC1=CC=C(CN(C)C)O1)=C/[N+]([O-])=O	314.46	10	8.451	86.26	111.56
Betaxolol	CC(C)NCC(O)COC1=CC=C(CCOCC2CC2)C=C1	307.48	11	7.331	50.72	50.72
Pravastatin	CC[C@H](C)C(=O)O[C@H]1C[C@H](O)C=C2C=C[C@H](C)[C@H](	424.59	11	9.388	124.29	124.29

	CC[C@@H](O)C[C@@H](O)CC(O)=O)[C@@H]12					
Cefuroxime Axetil	CO\N=C(/C(=O)N[C@H]1[C@H]2SCC(COC(N)=O)=C(N2C1=O)C(=O)OC(C)OC(C)=O)C1=CC=CO1	510.53	12	9.096	189.06	214.36
Lisinopril	NCCCC[C@H](N[C@@H](CCC1=CC=CC=C1)C(O)=O)C(=O)N1CCC[C@H]1C(O)=O	405.55	12	9.582	132.96	132.96
Verapamil	COC1=CC=C(CCN(C)CCCC(C#N)(C(C)C)C2=CC=C(OC)C(OC)=C2)C=C1OC	454.67	13	10.501	63.95	63.95

Table S6: selection of 17 rigid compounds and calculation of TPSA and 3D PSA methods.

	TPSA/3D PSA	TPSA					3D PSA					
	Atom selection	N, O, H			N, O, S, P, H		N, O, H		N, O, S, P, H			
Compound	SMILES	Molinspiration	TPSA (NO)	Marvin_no_SP	Dragon Tot	Marvin default	Pymol no	Pymol noh	Vega	Pymol nosp	pymol nosph	PSA Clark 1999
Acetaminophen	Oc1ccc(NC(=O)C)cc1	49.33	49.33	49.33	49.33	49.33	33.52	51.20	51.25	33.52	51.20	58.50
Bromazepam	Brc1cc2c(NC(=O)CN=C2c2ncccc2)cc1	54.35	54.35	54.35	54.35	54.35	39.64	48.22	47.51	39.64	48.22	57.40
Caffeine	O=C1N(C)C(=O)N(c2ncn(c12)C)C	61.84	61.82	58.44	61.82	58.44	51.72	51.72	50.62	51.72	51.72	59.20
Chlorothiazide	Clc1cc2NC=NS(=O)(=O)c2cc1S(=O)(=O)N	118.70	118.69	118.69	135.45	135.45	95.71	122.38	134.57	108.33	135.01	134.30

Coumarin	O1c2c(C=CC1=O)cccc2	30.21	30.21	26.30	30.21	26.30	28.40	28.40	28.50	28.40	28.40	33.90
Diazepam	Clc1cc2c(N(C)C(=O)CN=C2c2ccccc2)cc1	32.67	32.67	32.67	32.67	32.67	28.97	28.97	28.42	28.97	28.97	34.50
Foscarnet	P(=O)([O-])([O-])C(=O)[O-]	103.31	103.32	102.32	113.13	113.13	94.79	94.79	113.69	102.54	114.20	117.30
Hydrochlo-rothiazide	Clc1cc2NCNS(=O)(=O)c2cc1S(=O)(=O)N	118.36	118.36	118.36	135.12	135.12	92.09	127.72	139.06	104.72	140.34	135.20
Lamotrigine	Clc1c(cccc1Cl)-c1nnc(nc1N)N	90.72	90.71	90.71	90.71	90.71	48.55	81.60	83.31	48.55	81.60	96.40
Lormetaze-pam	Clc1ccccc1C1=NC(O)C(=O)N(c2c1cc(Cl)cc2)C	52.90	52.90	52.90	52.90	52.90	40.50	49.44	50.63	40.50	49.44	55.90

Nordiaze-pam	Clc1cc2c(NC(=O)CN=C2c2ccccc2)cc1	41.46	41.46	41.46	41.46	41.46	32.29	40.89	40.60	32.29	40.89	47.50
Oxazepam	Clc1cc2c(NC(=O)C(O)N=C2c2ccccc2)cc1	61.69	61.69	61.69	61.69	61.69	44.14	62.01	61.73	44.14	62.01	55.60
Phenazone	O=C1N(N(C)C(=C1)C)c1ccccc1	26.94	26.93	23.55	26.93	23.55	24.52	24.52	25.96	24.52	24.52	28.00
Progester-one	O=C1CC[C@@]2([C@@H]3[C@H]([C@@H]4CC[C@H](C(=O)C)[C@]4(CC3)C)CCC2=C1)C	34.14	34.14	34.14	34.14	34.14	33.50	33.50	33.51	33.50	33.50	41.20
Salicylic Acid	O=C(O)c1ccccc1O	57.53	57.53	57.53	57.53	57.53	39.90	58.62	57.34	39.90	56.24	62.50
Theophyl-line	O=C1N(C)C(=O)N(	72.69	72.68	69.30	72.68	69.30	57.16	66.21	63.90	57.16	66.21	74.70

	c2nc[nH]c12)C											
Testos-terone	O=C1CC[C@@]2([C@@H]3[C@H]([C@@H]4CC[C@H](O)[C@]4(CC3)C)CCC2=C1)C	37.30	37.30	37.30	37.30	37.30	30.26	39.31	39.27	30.26	39.31	43.40

Table S7: Pearson's correlation matrix for TPSA and 3D PSA methods.

	TPSA					3D PSA					
	N, O, H			N, O, S, P, H		N, O, H		N, O, S, P, H			
	Molinspiration	Dragon NO	Marvin no SP	Dragon tot	Marvin default	Pymol no	Pymol noh	PSA Literature	Vega	Pymol nosp	Pymol nosph
Molinspiration		1.00	1.00	0.99	0.99	0.90	0.97	0.98	0.97	0.89	0.97
TPSA(NO)	1.00		1.00	0.99	0.99	0.90	0.97	0.98	0.97	0.89	0.97
Marvin_no_SP	1.00	1.00		0.99	0.99	0.89	0.98	0.98	0.97	0.88	0.97
TPSA(Tot)	0.99	0.99	0.99		1.00	0.93	0.98	0.99	0.99	0.93	0.99
Marvin_default	0.99	0.99	0.99	1.00		0.92	0.99	0.99	0.99	0.92	0.99
pymol_no	0.90	0.90	0.89	0.93	0.92		0.88	0.91	0.92	1.00	0.93

pymol_noh	0.97	0.97	0.98	0.98	0.99	0.88		0.98	0.99	0.89	0.99
PSA/Å2	0.98	0.98	0.98	0.99	0.99	0.91	0.98		0.98	0.91	0.98
Vega (0)	0.97	0.97	0.97	0.99	0.99	0.92	0.99	0.98		0.93	1.00
pymol_nosp	0.89	0.89	0.88	0.93	0.92	1.00	0.89	0.91	0.93		0.94
pymol_nosph	0.97	0.97	0.97	0.99	0.99	0.93	0.99	0.98	1.00	0.94	

Table S8: Fitting curve slope for correlated TPSA/3D PSA pairs.

	N, O, H		N, O, S, P, H		
TPSA	**pymol_no**	**pymol_noh**	**Vega**	**pymol_nosp**	**pymol_nosph**
Molinspiration, Dragon (NO), Marvin (no S, P)	0.71	1.07	1.19	0.85	1.2
Dragon (tot), Marvin default	0.65	0.88	0.97	0.77	0.98

Table S9: Fitting curve Y intercepts for correlated TPSA/3D PSA pairs.

	N, O, H		N, O, S, P, H		
TPSA	**pymol_no**	**pymol_noh**	**Vega**	**pymol_nosp**	**pymol_nosph**
Molinspiration, Dragon (NO), Marvin (no S, P)	1.65	-3.39	-10.5	-4.6	10.7
Dragon (tot), Marvin default	6.04	3.08	-1.5	2	-1.9

Table S10: Comparable PSA/TPSA methods according to our study.

TPSA method	3D PSA method
Molinspiration, Dragon (NO), Marvin (no S, P)	PyMol_noh (in-house)
Marvin, Dragon (tot)	Vega (Radius 0 Å), PyMol_nosph (in-house)

Table S11: Relevant angles and distances for IMHB detection with sp2-O acceptors (HBA). Color code for chemical requirements; green: sufficient, yellow: improbable with condition relaxation, red: critical.

**Molecule**	**Category**	**R (Å) <**	**ν**	**θ (º)>**
Ph-NH_2	HBD (Chimera)	3.3	100-146	145
R-NH_2	HBD (Chimera)	3.3	90-128	150
Pomalidomide	HBD /HBA	**2.5**	**78**	**120**

Table S12: Relative 3D PSA differences in the generated conformers ensembles

		Difference %		
**Method**	**Compound**	**Δ Max p - Min np* %**	**Δ Average p - Average np* %**	**Δ Median p - Median np* %**
**CS**	**Pomalidomide**	1.3	0.4	0.4
	**Saquinavir**	14.6	4.8	4.2
	**CMP 98**	12.3	6.8	7.1
**MD**	**Pomalidomide**	9.5	0.6	0.6
	**Saquinavir**	17.5	3.7	4.1
	**CMP 98**	15.8	5.1	5.1
**SMD tunneling**	**Pomalidomide**	9.2	0.6	0.6
	**Saquinavir**	20.8	5.4	5.7
	**CMP 98**	17.0	3.9	3.9

Table S13: Relative Rgyr differences in the generated conformers ensembles

Method	Compound	Difference		
		Δ Max p - Min np* %	Δ Average p - Average np* %	Δ Median p - Median np* %
CS	Pomalidomide	4.9	0.31	0.3
	Saquinavir	21.0	-3.98	-2.9
	CMP 98	12.8	-1.00	-0.9
MD	Pomalidomide	7.4	0.22	0.2
	Saquinavir	24.1	4.11	3.8
	CMP 98	57.7	8.65	4.9
SMD tunneling	Pomalidomide	7.9	0.25	0.3
	Saquinavir	30.9	9.86	9.7
	CMP 98	56.7	-9.20	-12.6
